# A thermodynamically consistent model of the post-translational Kai circadian clock

**DOI:** 10.1371/journal.pcbi.1005415

**Published:** 2017-03-15

**Authors:** Joris Paijmans, David K. Lubensky, Pieter Rein ten Wolde

**Affiliations:** 1 FOM Institute AMOLF, Amsterdam, The Netherlands; 2 Department of Physics, University of Michigan, Ann Arbor, Michigan, United States of America; Rice University, UNITED STATES

## Abstract

The principal pacemaker of the circadian clock of the cyanobacterium *S. elongatus* is a protein phosphorylation cycle consisting of three proteins, KaiA, KaiB and KaiC. KaiC forms a homohexamer, with each monomer consisting of two domains, CI and CII. Both domains can bind and hydrolyze ATP, but only the CII domain can be phosphorylated, at two residues, in a well-defined sequence. While this system has been studied extensively, how the clock is driven thermodynamically has remained elusive. Inspired by recent experimental observations and building on ideas from previous mathematical models, we present a new, thermodynamically consistent, statistical-mechanical model of the clock. At its heart are two main ideas: *i*) ATP hydrolysis in the CI domain provides the thermodynamic driving force for the clock, switching KaiC between an active conformational state in which its phosphorylation level tends to rise and an inactive one in which it tends to fall; *ii*) phosphorylation of the CII domain provides the timer for the hydrolysis in the CI domain. The model also naturally explains how KaiA, by acting as a nucleotide exchange factor, can stimulate phosphorylation of KaiC, and how the differential affinity of KaiA for the different KaiC phosphoforms generates the characteristic temporal order of KaiC phosphorylation. As the phosphorylation level in the CII domain rises, the release of ADP from CI slows down, making the inactive conformational state of KaiC more stable. In the inactive state, KaiC binds KaiB, which not only stabilizes this state further, but also leads to the sequestration of KaiA, and hence to KaiC dephosphorylation. Using a dedicated kinetic Monte Carlo algorithm, which makes it possible to efficiently simulate this system consisting of more than a billion reactions, we show that the model can describe a wealth of experimental data.

## Author summary

Circadian clocks are biological timekeeping devices with a rhythm of 24 hours in living cells pertaining to all kingdoms of life. They help organisms to coordinate their behavior with the day-night cycle. The circadian clock of the cyanobacterium *Synechococcus elongatus* is one of the simplest and best characterized clocks in biology. The central clock component is the protein KaiC, which is phosphorylated and dephosphorylated in a cyclical manner with a 24 hr period. While we know from elementary thermodynamics that oscillations require a net turnover of fuel molecules, in this case ATP, how ATP hydrolysis drives the clock has remained elusive. Based on recent experimental observations and building on ideas from existing models, we construct the most detailed mathematical model of this system to date. KaiC consists of two domains, CI and CII, which each can bind ATP, yet only CII can be phosphorylated. Moreover, KaiC can exist in two conformational states, an active one in which the phosphorylation level tends to rise, and an inactive one in which it tends to fall. Our model predicts that ATP hydrolysis in the CI domain is the principal energetic driver of the clock, driving the switching between the two conformational states, while phosphorylation in the CII domain provides the timer for the conformational switch. The coupling between ATP hydrolysis in the CI domain and phosphorylation in the CII domain leads to novel testable predictions.

## Introduction

Circadian clocks, which allow organisms to anticipate changes in day and night, are a fascinating example of biological rhythms. One of the most studied and best characterized models of circadian oscillations is the cyanobacterium *Synechococcus elongatus*. It is now known that it combines a protein synthesis cycle [1–3] with a protein phosphorylation cycle [4], and in 2005 the latter was reconstituted in the test tube [5]. This stimulated a detailed characterization of its design principles, in a fruitful collaboration between experiments and modeling [6–17]. Yet, it has remained an open question how the clock is driven thermodynamically.

The central components of the protein phosphorylation cycle are three proteins, KaiA, KaiB, and KaiC. KaiC forms a homohexamer with two phosphorylation sites per monomer, serine 431 and threonine 432 [3], which are phosphorylated and dephosphorylated in a well-defined temporal order [8, 18]. KaiA stimulates phosphorylation [19–21], while KaiB negates the effect of KaiA [20–23]. Each individual hexamer can be thought of as a tiny oscillator, going through a phosphorylation cycle in roughly 24 hours. To generate macroscopic oscillations, the individual oscillators have to tick in phase with each other. Due to the inherent noise in biochemical reactions, however, the phosphorylation cycles of the respective hexamers will inevitably run out of phase when there is no mechanism to synchronize them. Modeling in combination with experiments indicate that the oscillations of the individual hexamers are synchronized via the mechanism of differential affinity: When the majority of the KaiC hexamers is in the dephosphorylation phase of the cycle, there are front runners that have already completed their cycle, and are now ready to be phosphorylated again. Phosphorylation requires KaiA, however, and the limited supply of KaiA is sequestered by the laggards that are still in the dephosphorylation phase of the cycle, where they bind KaiA very strongly together with KaiB. By taking KaiA away from the front runners, the laggards thus force the front runners to slow down, narrowing the distribution of phosphorylation states across hexamers [8, 9]. This is the essence of the mechanism of differential affinity and it appears to be active not only during the dephosphorylation phase of the cycle [8, 9], but also during the phoshorylation phase [9, 24]. Monomer exchange between hexamers [6], observed in experiments [11], is an alternative synchronization mechanism. However, theoretical studies by us and others suggest that monomer exchange is not critical for stable oscillations [8–10].

While it is clear that the clock is driven by the turnover of ATP [25, 26], how fuel turnover drives the phosphorylation oscillations is still unclear. In previous models [9, 24, 27], phosphorylation is driven by ATP hydrolysis, while dephosphoryation proceeds via the spontaneous release of the phosphate groups from the threonine and serine residues. Intriguingly, however, recent experiments have revealed that during the dephosphorylation phase of the clock, ATP is regenerated [28, 29]: the phosphate groups on the serine and threonine residues are transferred back to ADP. If phosphorylation and dephosphorylation do not cause any net turnover of ATP, what then drives the clock? Clocks are necessarily dissipative, entailing a net turnover of fuel molecules per cycle.

KaiC consists of two highly homologous domains, called the CI and the CII domain [30]. Both domains can bind and hydrolyze ATP [31, 32], but only the CII domain can be phosphorylated [33]. The ATP regeneration experiments indicate that the phosphorylation and dephosphorylation of CII proceeds via the transfer of phosphate groups between the threonine/serine residues and the nucleotide bound to CII [28, 29], leaving open the possibility that there is no net turnover of ATP on the CII domain.

Here, we argue that the hydrolysis of ATP in the CI domain is the principal energetic driver of the clock. We present a new mathematical model of the post-translational Kai circadian clock in *S. elongatus*, which is based on the idea that ATP hydrolysis in CI drives a conformational switch of KaiC. Previously, it has been predicted that ATP hydrolysis plays an important role in driving conformational transitions [27, 34] and that these transitions are vital to generating the oscillations [9, 24], predictions that have found experimental support [27, 34–38]. Our model, however, goes farther, predicting that ATP hydrolysis in the CI domain is the place where detailed balance must be broken in order to generate sustained oscillations. Our model is inspired by that of Van Zon *et al*. [9]. KaiC switches between an active conformation in which the phosphorylation level tends to rise, and an inactive one in which it tends to fall [9]. The model describes how KaiA binds to the CII domain of KaiC in the active conformation, and how KaiA can then drive phosphorylation by acting as a nucleotide exchange factor [39], stimulating the exchange of ADP for ATP. The model predicts that as the phosphorylation level in the CII domain rises, the release of ADP from CI slows down. The ADP-bound state makes the inactive conformation of KaiC more stable, causing the hexamer to flip to the inactive state and triggering dephosphorylation. In our model, ATP hydrolysis in the CI domain thus provides the thermodynamic driving force for the oscillations, while the phosphorylation in the CII domain provides the timer for the hydrolysis in the CI domain.

While the coupling between ATP hydrolysis in the CI domain and phosphorylation in the CII domain is the main feature of the new model, leading to novel testable predictions, the model can also describe a wealth of additional experimental data. The differential affinity of KaiA for the different KaiC phosphoforms naturally explains the characteristic sequence in which the threonine and serine sites are phosphorylated [8, 18]. In addition, while retention of ADP in the CI domain triggers a switch between the active and inactive KaiC conformations, the binding of KaiB to CI stabilizes the inactive state further, and leads, as in previous models [8, 9, 24], to the sequestration of KaiA, necessary for synchronizing the oscillations. The model predicts that the slow binding of KaiB, as observed experimentally [40], introduces a delay between the moment that a given KaiC hexamer reaches its point of maximum phosphorylation, and hence no longer needs KaiA to progress along the phosphorylation cycle, and the moment that the same KaiC actually sequesters KaiA. In our model, this delay is essential because it allows the laggards to reach the top of the cycle before the front runners take away KaiA. Although our model predicts that ATP hydrolysis in CII is sufficient for generating cycles in *individual* hexamers, hydrolysis in CI is necessary for creating macroscopic oscillations: ATP hydrolysis in CI drives the conformational switch to the inactive state, which leads to the synchronization of the hexamers through the sequestration of KaiA. Lastly, the model can explain the experimental observation that the oscillation period is robust to variations in steady-state ATP/ADP levels, while the system can be entrained by transient changes in this ratio, which is one of the important mechanisms for coupling the clock to light [41].

## Model

### Model overview

**Fig 1:**
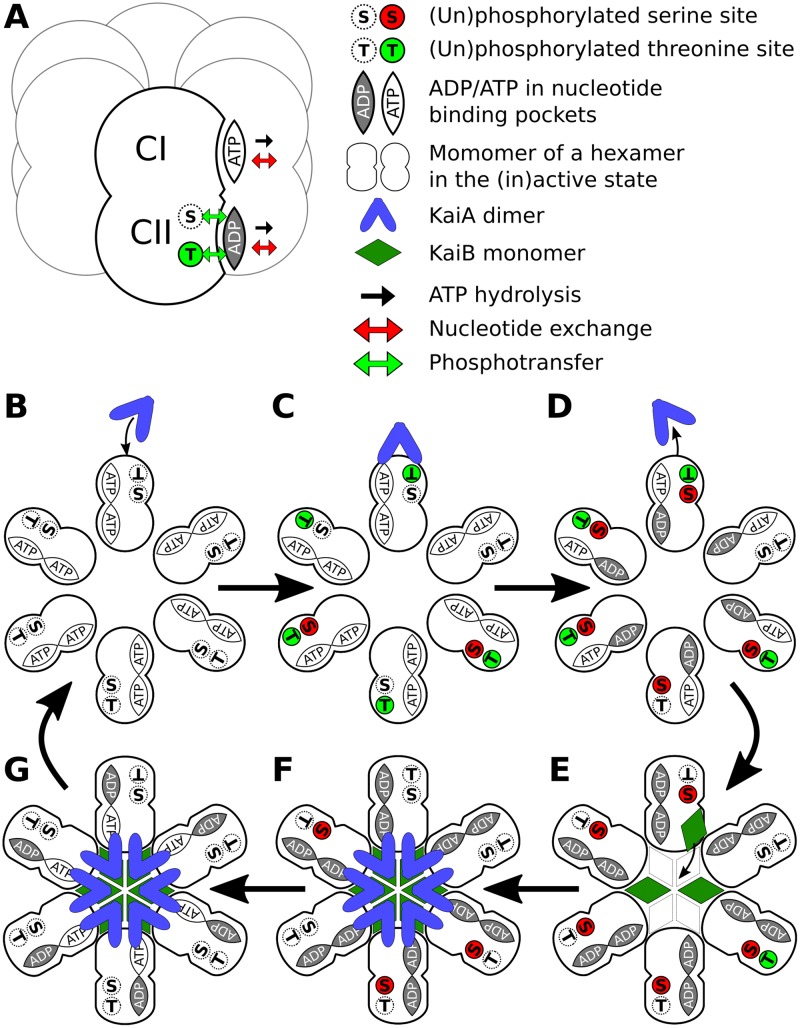
Overview of the model and of how a hexamer progresses through its cycle. (A) KaiC hexamer with one monomer highlighted. Each monomer consists of a CI and a CII domain. Both domains can bind nucleotides, but only the CII domain can be phosphorylated, at a serine and a threonine residue. In the cartoon shown, CI has ATP bound while CII has ADP bound; in CII, the threonine residue is phosphorylated whereas the serine residue is not. Arrows indicate allowed reactions: ATP bound to either domain can be hydrolyzed to ADP, and bound ADP and ATP can be exchanged. The serine and threonine residues in the CII domain can be phosphorylated through phospho-transfer from a bound ATP and de-phosphorylated through phospho-transfer from the residue to a bound ADP. Panels B-G show a top view of the hexamer of panel A folded open for reasons of clarity; in each monomer, the CI domain is towards the center and the CII domain towards the outside. They illustrate how the hexamer traverses a typical phosphorylation cycle in a buffer with ATP. Starting with an unphosphorylated hexamer in the active state, the nucleotide exchange rate of the binding pockets of the CI domain with the bulk will be high such that they have predominantly ATP bound. A single KaiA dimer can bind to the CII domain, which enhances the nucleotide exchange rate in all the CII binding pockets such that the probability that ATP is bound increases. ATP in CII drives phosphorylation, where first the T sites and later the S sites are phosphorylated (C). When more serine sites than threonine sites are phosphorylated (D), the nucleotide exchange rate in the CI domain will drop and any remaining ATP in the binding pockets of CI will be hydrolyzed so that most CI domains have ADP bound. ADP in the CI domain will stabilize the inactive state, causing the hexamer to change conformation and allowing for KaiB monomers to slowly bind the CI domain (E). When six KaiB monomers are bound, the hexamer will sequester 6 KaiA dimers (F). Without KaiA available to bind to the CII domain, the nucleotide exchange rate in the binding pocket of the CII domain is low, and all ATP is hydrolyzed to ADP, allowing the hexamer to dephosphorylate. When no serine sites are phosphorylated, the nucleotide exchange rate in the binding pocket of the CI domain increases again, releasing the ADP and replacing it with ATP (G). The hexamer then flips back to the active state and releases the sequestered KaiA and KaiB.

Our model of the in-vitro Kai circadian clock [5] builds on the hexamer model developed by Van Zon and coworkers [9]. But in contrast to that model, and following the models developed by Rust *et al*. [8, 24, 27], it explicitly keeps track of the two phosphorylation sites on each of the monomers, as well as their nucleotide binding states. The purpose of this section is to give an overview of the new model and its state variables, and to provide background information on ideas from previous models and their experimental justification. The new ingredients of the model, as well as their experimental movitation, are discussed only briefly; they are discussed in much more detail in the sections below. A cartoon of the model, illustrating the states KaiC can be in and how the system progresses through the cycle, is shown in Fig 1.

#### KaiC monomers

Our model follows the phosphorylation and nucleotide-binding state of each of the six monomers inside a hexamer. Each monomer consists of two domains: The CI and the CII domain. The CII domain has two phosphorylation sites, the threonine and the serine site, resulting in four different phosphorylation states [8, 18]: unphosphorylated (U), phosphorylated only on serine (S), phosphorylated only on threonine (T), and phosphorylated on both serine and threonine (D). Furthermore, both the CI and CII domains have a nucleotide binding pocket which can be in one of two possible states: Either there is adenosine triphosphate (ATP) or adenosine diphosphate (ADP) bound to it. The unbound state is ignored, because nucleotide binding is much faster than nucleotide dissociation, as described below. The state variables of the monomers are given in Table 1.

**Table 1:** Monomer and hexamer state variables, with possible values. Variables *n*^CI·KaiA^ and *n*^CII·KaiA^ count the number of KaiA dimers bound to the CI and CII domain, respectively, and *n*^CI·KaiB^ counts the number of KaiB monomers bound to CI.

Monomer		Hexamer	
Variable	States	Variable	States
Phosphorylation	U,T,D,S	Conformation	Active/Inactive
CI Binding pocket	ATP,ADP	*n*^CII·KaiA^	0, 1
CII Binding pocket	ATP,ADP	*n*^CI·KaiB^	0−6
		*n*^CI·KaiA^	0−6

In the next sections, we describe in detail how ATP hydrolysis in CI drives the conformational switch of KaiC and how phosphorylation in CII controls hydrolysis in CI.

#### KaiC hexamers

Van Zon *et al*. postulated that KaiC can be in either an active (A) or inactive (I) conformation [9]. Experiments probing the exposure of the C terminal tails of KaiC and the stacking interactions between the CI and CII domains indeed provide evidence that KaiC can exist in multiple conformational states [34, 35, 37, 38]. We follow Van Zon *et al*., and assume in the spirit of the Monod-Wyman-Changeux (MWC) model [42] that the CI and CII domains of all the monomers in a hexamer switch conformation in concert, such that we can speak of the hexamer as either being in the active or inactive state. Following Van Zon *et al*. our model does not include monomer exchange, which does not appear to be essential [8–10].

#### KaiB binding

The phosphorylation behavior of KaiC in the presence of KaiB, but not KaiA, is highly similar to that of KaiC alone [8, 21, 22]. This observation indicates that KaiB does not directly affect the phosphorylation and dephosphorylation rates. Following Van Zon *et al*. [9], we assume instead that KaiB plays the following dual role: *i*) KaiB binding increases the stability of the inactive state by binding to the CI domain of inactive KaiC; experimental observations support the idea that the binding of KaiB to KaiC depends on the conformational state of KaiC [34, 37, 38]; moreover, the experiments show that KaiB binding peaks in the dephosphorylation phase of the cycle, when KaiC is in the inactive conformational state [8, 43, 44]; *ii*) KaiB associated with the CI domain of inactive KaiC strongly binds the limiting pool of KaiA, thereby sequestering it. In our model, we do not explicitly keep track of the KaiB concentration. Recent experiments show that KaiB binds KaiC as a monomer [38], but is energetically most stable as a tetramer [40]. Because of the equilibrium between the terameric and monomeric state of KaiB, the tetrameric state will act as a reservoir stabilizing the KaiB monomer concentration, making the latter less dependent on the total KaiB concentration [38]. Moreover, as predicted by Van Zon *et al*. [9], as long as KaiC can bind enough KaiB to sequester KaiA effectively, the concentration of KaiB does not affect the amplitude and period of the oscillations [45]. We therefore do not explicitly model the concentration of KaiB, but rather include it in the definition of the effective rate constant for KaiB-KaiC binding.

#### KaiA binding

Experiments have unambiguously demonstrated that KaiA stimulates the phosphorylation of KaiC [3, 20–22]. Moreover, they indicate that in the absence of KaiB, KaiA binds to the CII domain [46]. Inspired by the recent observation that KaiA acts as a nucleotide exchange factor [39], our new model describes how KaiA bound to CII is able to drive phosphorylation by controlling the nucleotide exchange rate. In the presence of KaiB, KaiA can also bind to the CI domain of inactive KaiC [34]. We do keep track of the KaiA dimers in the solution, and explicitly model their binding to the CII domain and their sequestration on the CI domain via KaiB. The interactions are always described as bimolecular reactions between KaiC hexamers and KaiA dimers or KaiB monomers. For simplicity, and lack of experimental evidence suggesting otherwise, the binding of KaiA or KaiB to KaiC always affects all monomers in the hexamer equally. In our model, a single KaiA dimer can bind to the CII domain of the hexamer, six KaiB monomers can bind to the CI domain of a KaiC hexamer and six KaiA dimers can, in turn, be sequestered by the CI domains of the hexamer in the inactive state.

#### State variables and parameters

The state variables describing the hexamer and possible values are summarized in Table 1. The parameters of the model were obtained by fitting the predictions of the model to experimental data, as explained in the sections below. In this procedure, the parameters were “hand tuned”—we did not follow a systematic, formal, fitting procedure.

#### Article overview

We have split the explanation of the full model into two parts: First we give a detailed description of the phosphorylation dynamics in the Kai system which primarily concerns the CII domain of KaiC and its interaction with KaiA. In the next part, we describe the power cycle in the CI domain, the connection between the CI and CII domain and the binding and unbinding kinetics of KaiB and the subsequent sequestration of KaiA by the CI domain. Then we present the results for the model, again split into two sections: One relating to the phosphorylation dynamics and one to the power cycle in the CI domain.

### Model of the KaiC phosphorylation dynamics

Here we give a detailed description of how the phosphotranfer reactions, the ratio of ATP to ADP in the binding pockets and differential affinity of KaiA together give rise to the ordered phosphorylation of the serine and threonine sites in the CII domain. In steps, we present the foundations of our model together with the experimental results that underlie it and give a detailed mathematical description of the resulting free energies and reaction rates.

#### Phosphorylation and dephosphorylation only occur via phosphotransfer with ATP and ADP

Recent experiments unexpectedly showed that during the dephosphorylation of KaiC, the inorganic phosphate group on the serine and threonine sites of KaiC is transfered to the ADP in the binding pocket of the CII domain, effectively regenerating the ATP that was used for phosphorylation [28, 29]. We hypothesize that in our model, dephosphorylation without a nucleotide as an intermediate does not occur. Therefore, the phosphotransfer reactions are the only pathways for phosphorylation and dephosphorylation of KaiC,

$$\begin{array}{ll} \text{U}\cdot \text{ATP}\overset{k_{\text{UT}}^{0}}{\underset{k_{\text{TU}}^{0}}{⇌}}\text{T}\cdot \text{ADP,} & \text{T}\cdot \text{ATP}\overset{k_{\text{TD}}^{0}}{\underset{k_{\text{DT}}^{0}}{⇌}}\text{D}\cdot \text{ADP,}\\ \text{U}\cdot \text{ATP}\overset{k_{\text{US}}^{0}}{\underset{k_{\text{SU}}^{0}}{⇌}}\text{S}\cdot \text{ADP,} & \text{S}\cdot \text{ATP}\overset{k_{\text{SD}}^{0}}{\underset{k_{\text{DS}}^{0}}{⇌}}\text{D}\cdot \text{ADP}. \end{array}$$ (1)

Here, U,T,D and S correspond to the phosphorylation state of the monomer and ATP and ADP denote the state of the CII nucleotide binding pocket. $k_{\text{XY}}^{0}$ are the phosphotransfer rate constants when KaiA is not bound to CII. Since these rates are independent of the state of the other monomers, the monomers in a hexamer are phosphorylated in a random order. As is clear from Eq 1, the phosphorylation dynamics critically depends on the state of the nucleotide binding pocket of the CII domain: With ATP in the binding pocket, KaiC can only be phosphorylated and with ADP in the binding pocket KaiC can only be dephosphorylated. Therefore, we explicitly keep track of the state of the nucleotide binding pocket adjacent to the serine and threonine sites of each monomer.

#### KaiA acts as a nucleotide exchange factor on the binding pockets of the CII domain

*Nucleotide exchange rate in absence of KaiA*. Since the nucleotide binding rates are much faster than the dissociation rates, the unbound state can be neglected [28, 31, 32], and the nucleotide binding pocket will alternate only between the ATP and ADP bound state, both on CI and CII. Assuming that the association rates for ATP and ADP are diffusion limited and similar, the dynamics of nucleotide exchange will solely be governed by the nucleotide dissociation rates, which for the CII domain, discussed here, are denoted by $k_{\text{off}}^{\text{CII}\cdot \text{ATP}}$ and $k_{\text{off}}^{\text{CII}\cdot \text{ADP}}$, respectively. Because measured nucleotide exchange rates are very low when KaiA is not bound to the CII domain [28, 39], in our model the dissociation rates of ATP and ADP are non-zero only when KaiA is bound to the CII domain. Next to nucleotide exchange, ATP can also be converted to ADP via hydrolysis with a rate $k_{\text{hyd}}^{\text{CII}}$ [25, 28]. Since hydrolysis is a strong downhill reaction under experimental conditions, we neglect the reverse reaction of the hydrolysis pathway, such that there is no ATP production by spontaneous binding of a phosphate group to ADP. The relative affinity between ATP and ADP for the nucleotide binding pocket of the CII domain is now given by $K_{\text{ATP}/\text{ADP}}^{\text{CII}}=K_{d}^{\text{CII}\cdot \text{ATP}}/K_{d}^{\text{CII}\cdot \text{ADP}}=k_{\text{off}}^{\text{CII}\cdot \text{ATP}}/k_{\text{off}}^{\text{CII}\cdot \text{ADP}}$. Assuming that the nucleotide exchange and hydrolysis pathways are independent, we can simply add their reaction rate constants, such that the rates for changing between the ATP and ADP bound states become

$$\begin{array}{ccc} k_{\text{ATP}\to \text{ADP}}^{\text{CII}} & = & k_{\text{hyd}}^{\text{CII}}+\left(1-\alpha_{\text{ATP}}^{}\right)\;k_{\text{off}}^{\text{CII}\cdot \text{ATP}}, \end{array}$$ (2)

$$\begin{array}{ccc} k_{\text{ADP}\to \text{ATP}}^{\text{CII}} & = & \alpha_{\text{ATP}}^{}\;k_{\text{off}}^{\text{CII}\cdot \text{ADP}}. \end{array}$$ (3)

Here, *α*_ATP_ is the fraction, [ATP]/([ATP] + [ADP]), of ATP nucleotides in the solution. The rate from ATP to ADP (Eq 2) is the sum of the hydrolysis rate plus the rate of dissociating ATP times the probability of immediately binding an ADP, which due to the equal association rates for ADP and ATP binding, is simply given by the fraction of ADP in the bulk (1 − *α*_ATP_). The reverse rate (Eq 3) is given by the rate of dissociating ADP times the probability, *α*_ATP_, of binding an ATP nucleotide.

*KaiA speeds up nucleotide exchange on CII domain*. It is well known that KaiA stimulates the phosphorylation of KaiC [3, 20], and that without KaiA, KaiC dephosphorylates [21, 22]. Recent experiments showed that KaiA increases the fraction of ATP in the nucleotide binding pockets and thereby stimulates phosphorylation [39, 47]. Without KaiA bound to the CII domain, the exchange rates between the nucleotide binding pocket and the bulk are very low, such that eventually all ATP molecules bound to the binding pockets of the CII domain are hydrolyzed [28, 39]. Given these observations, in our model KaiA will act as a nucleotide exchange factor increasing the dissociation rates of both ATP and ADP in equal amounts, such that the relative affinity, $K_{\text{ATP}/\text{ADP}}^{\text{CII}}$, is unchanged.

We model the interaction between KaiA and the CII domain on three simplifying assumptions: First, only one KaiA dimer can bind to the CII domain of a hexamer, although a higher stoichiometry has been observed [48, 49]. Second, when KaiA is bound to CII, it enhances the nucleotide exchange rates in all the monomer binding pockets equally. Third, when KaiA is not bound, the nucleotide dissociation rates are zero. Therefore, the nucleotide dissociation rates given in Eqs 2 and 3, are given by $k_{\text{off},\text{KaiA}}^{\text{CII}\cdot \text{ADP}}$ $K_{\text{ATP}/\text{ADP}}^{\text{CII}}$ and $k_{\text{off},\text{KaiA}}^{\text{CII}\cdot \text{ADP}}$, respectively, when KaiA is bound to CII, and equal zero when KaiA is absent. The hydrolysis rate, $k_{\text{hyd}}^{\text{CII}}$, does not depend on whether KaiA is bound to the CII domain, in the interest of simplicity.

**Fig 2:**
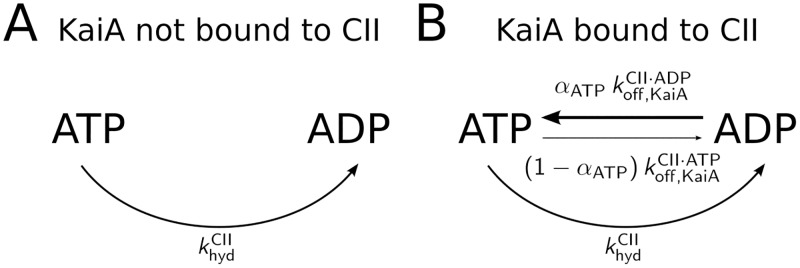
KaiA regulates the fraction of ATP in the CII binding pockets by increasing the nucleotide dissociation rates, in combination with the ATPase activity in the CII domain. A) Without KaiA bound to CII, the nucleotide dissociation rates are identically zero, and there is no nucleotide exchange with the bulk. Since ATP is hydrolyzed at a constant rate, $k_{\text{hyd}}^{\text{CII}}$, eventually all the binding pockets will be occupied by ADP. B) When KaiA is bound to the CII domain, it increases the dissociation rates of ADP and ATP, $k_{\text{off}}^{\text{CII}\cdot \text{ATP}}$ and $k_{\text{off}}^{\text{CII}\cdot \text{ADP}}$, respectively, while leaving the affinities for ATP and ADP unchanged. Now, ADP in the CII domain is replaced by ATP at a rate that is faster than that at which ATP is hydrolyzed; this indeed increases the fraction of ATP in the binding pockets. For simplicity, we assumed equal diffusion limited association rates for ATP and ADP, such that the probability of binding ATP after ADP has dissociated is equal to *α*_ATP_. The rate of the reverse pathway is proportional to the bulk ADP fraction, 1 − *α*_ATP_.

*KaiA stimulates phosphorylation by speeding up nucleotide exchange*. Fig 2 illustrates how KaiA can enhance the ATP fraction in the nucleotide binding pocket of the CII domain. KaiA does not change the affinity of CII for ATP and ADP, and hence also leaves their relative affinity unchanged. However, it does increase the binding and unbinding rates with the same magnitude. Moreover, the binding of the nucleotides is coupled to the non-equilibrium process of ATP hydrolysis, which breaks detailed balance. The result is that in the absence of KaiA, the hydrolysis rate dominates over the nucleotide exchange rates, driving the binding pockets towards the ADP state. In the presence of KaiA, exchange rates are larger than the hydrolysis rate, and because these rates favor ATP over ADP, the binding pocket is predominantly bound to ATP when KaiA is present. By increasing the occupation of ATP of the binding pocket, KaiA not only enhances phosphorylation but also blocks dephosphorylation since there is no ADP. Blocking dephosphorylation, which was implicitly present in previous models [8, 9, 24], is important, as it prevents futile phosphorylation cycles.

#### Differential affinity: The affinity of KaiA for KaiC depends on phosphorylation state of KaiC

It was predicted from theoretical arguments [9], and later confirmed by experiments [24, 50, 51], that the affinity of KaiC for KaiA depends on the phosphorylation state of the hexamer. By measuring the phosphorylation speed of KaiC, starting with different initial phosphorylation levels, Rust *et. al*. observed that the rate of KaiC phosphorylation decreases as the fraction of S and D phosphorylated KaiC monomers increases [24]. This suggests that KaiA has a high affinity when KaiC is in the U and T state, and a low affinity when KaiC is in the S and D state, leading to the mechanism of differential affinity [9].

Differential affinity means that the binding and unbinding rates of KaiA to the CII domain of KaiC depend on the phosphorylation state of the hexamer. The observation that KaiA predominantly binds to CII during the phosphorylation phase of the cycle indicates that, in addition, KaiA has a higher affinity for the active conformational state. In our model, when all the monomers of an active hexamer are in the unphosphorylated U state, KaiA will bind and unbind with the rates $k_{\text{on},0}^{\text{CII}\cdot \text{KaiA}}$ and $k_{\text{off},0}^{\text{CII}\cdot \text{KaiA}}$, respectively. The subsequent phosphorylation of KaiC changes the binding free energy $\Delta G_{\text{bind}}^{\text{CII}\cdot \text{KaiA}}$: this indeed underlies the mechanism of differential affinity. Assuming each monomer adds linearly to $\Delta G_{\text{bind}}^{\text{CII}\cdot \text{KaiA}}$, the change in the binding free energy between KaiA and CII becomes

$$\begin{array}{c} \Delta G_{\text{bind}}^{\text{CII}\cdot \text{KaiA}}=\sum_{i=1}^{6}\delta g_{\text{bind}}^{\text{CII}\cdot \text{KaiA}}\left(X_{i}\right)+h_{\text{Inactive}}^{}\;\delta g_{A,I}^{\text{CII}\cdot \text{KaiA}}. \end{array}$$ (4)

Here, $\delta g_{\text{bind}}^{\text{CII}\cdot \text{KaiA}}\left(X_{i}\right)$ is the contribution of each monomer in phosphorylation state *X*_*i*_ ∈ {*U*, *T*, *D*, *S*} to the binding free-energy. KaiA bound to the CII domain stabilizes the active conformational state with a fixed free-energy difference $\delta g_{A,I}^{\text{CII}\cdot \text{KaiA}}$ and *h*_Inactive_ is an indicator function that is one when the hexamer is in the inactive state and zero otherwise. Note that the stabilization of the active state with respect to the inactive one does not depend on the phosphorylation state, and therefore the hexamer’s conformation will not affect the phosphotransfer dynamics [8, 27]. Given the effect of differential affinity on the binding free energy, $\Delta G_{\text{bind}}^{\text{CII}\cdot \text{KaiA}}$, detailed balance dictates that the association and dissociation rates of KaiA become, respectively,

$$\begin{array}{ccc} k_{\text{on}}^{\text{CII}\cdot \text{KaiA}} & = & k_{\text{on},0}^{\text{CII}\cdot \text{KaiA}}\text{exp}\left(-\left(1-\lambda \right)\;\Delta G_{\text{bind}}^{\text{CII}\cdot \text{KaiA}}\right) \end{array}$$ (5)

$$\begin{array}{ccc} k_{\text{off}}^{\text{CII}\cdot \text{KaiA}} & = & k_{\text{off},0}^{\text{CII}\cdot \text{KaiA}}\text{exp}\left(\lambda \;\Delta G_{\text{bind}}^{\text{CII}\cdot \text{KaiA}}\right). \end{array}$$ (6)

Assuming changes in binding free-energy have an equal effect on the association and dissociation rate, *λ* = 1/2.

#### KaiA binding changes the phosphotransfer rates

**Fig 3:**
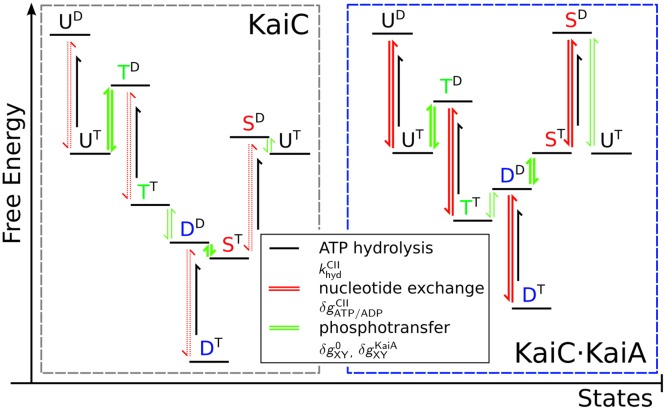
Proposed free energy landscape of the CII domain, without (left gray box) and with (right blue box) KaiA bound to the CII domain, for equal concentrations of ATP and ADP in the bulk. Superscripts denote the nucleotide bound state of CII. The landscape results from hand-tuning the phosphotransfer and nucleotide exchange parameters as well as the free-energy changes due to KaiA binding to give best agreement with the (de)phosphorylation assays performed in the SI of [27]. The free energy differences between phosphorylation states are defined by the detailed-balance relations, $\delta g_{\text{XY}}^{0}=-\log(k_{\text{XY}}^{0}/k_{\text{YX}}^{0})$ and $\delta g_{\text{XY}}^{\text{KaiA}}=-\log(k_{\text{XY}}^{\text{KaiA}}/k_{\text{YX}}^{\text{KaiA}})$ without and with KaiA bound to CII (connected by green arrows), respectively. Thick and thin green arrows indicate high and low phosphotransfer rates, respectively. Furthermore, $\delta g_{\text{ATP}/\text{ADP}}^{\text{CII}}=\log(K_{\text{ATP}/\text{ADP}}^{\text{CII}})$, gives the free-energy difference between nucleotide bound states (connected by red arrows). Note that the relative nucleotide affinity is independent of the KaiA-binding state or phosphorylation state. Dotted red arrows indicate the nucleotides exchange rates are identically zero when KaiA is not bound to CII, and solid red arrows in the right panel indicate high nucleotide exchange rates when KaiA is bound. The black arrows denote the irreversible hydrolysis of ATP. For hydrolysis, the magnitude of the arrow does not give the free energy change of the system since that involves the contribution from the phosphate release, which we do not take into account. Note that without hydrolysis, the total free-energy drop upon going through the cycle, *U*^T^ → *T*^D^ → *T*^T^ → *D*^D^ → *S*^T^ → *S*^D^ → *U*^T^, is zero.

We model phosphorylation and dephosphorylation, with and without KaiA, via a microscopically reversible phosphotranfer reaction. Then, as an inevitable consequence of differential affinity, detailed balance implies that the binding of KaiA must also influence the phosphotransfer rates [52]. We denote the free-energy difference between phosphorylation states X and Y, with X,Y∈ {U, T, D, S}, when KaiA is not bound to KaiC as $\delta g_{\text{XY}}^{0}$ and when KaiA is bound as $\delta g_{\text{XY}}^{\text{KaiA}}$. Detailed balance then implies that the difference between these two, $\delta g_{\text{XY}}^{0}-\delta g_{\text{XY}}^{\text{KaiA}}$, is equal to the change in the binding free-energy of KaiA that results from a change in the phosphorylation state X*_i_* to Y*_i_* of monomer *i*:

$$\begin{array}{ccc} \delta g_{\text{XY}}^{0}-\delta g_{\text{XY}}^{\text{KaiA}} & = & \Delta G_{\text{bind}}^{\text{CII}\cdot \text{KaiA}}\left(Y_{i}\right)-\Delta G_{\text{bind}}^{\text{CII}\cdot \text{KaiA}}\left(X_{i}\right) \end{array}$$ (7)

$$\begin{array}{ccc} & = & \delta g_{\text{bind}}^{\text{CII}\cdot \text{KaiA}}\left(Y\right)-\delta g_{\text{bind}}^{\text{CII}\cdot \text{KaiA}}\left(X\right), \end{array}$$ (8)

where $\delta g_{\text{XY}}^{0}=-\log(k_{\text{XY}}^{0}/k_{\text{YX}}^{0})$, with $k_{\text{XY}}^{0}$ given by Eq 1, and $\delta g_{\text{XY}}^{\text{KaiA}}=-\log(k_{\text{XY}}^{\text{KaiA}}/k_{\text{YX}}^{\text{KaiA}})$. The second line follows after substituting Eq 4 for $\Delta G_{\text{bind}}^{\text{CII}\cdot \text{KaiA}}$. The subscript *i* in Eq 7 is to emphasize that we compare hexamers that differ in the phosphorylation state of one of their monomers. Finally, we can write the phosphotransfer rate constants between states X and Y, for the situation where KaiA is bound to CII, as

$$\begin{array}{c} k_{\text{XY}}^{\text{KaiA}}=k_{\text{XY}}^{0}\;\exp\left(-\frac{1}{2}\left[\delta g_{\text{bind}}^{\text{CII}\cdot \text{KaiA}}\left(Y\right)-\delta g_{\text{bind}}^{\text{CII}\cdot \text{KaiA}}\left(X\right)\right]\right), \end{array}$$ (9)

and the reverse reaction rate by interchanging labels X and Y. This equation indeed shows that the phosphotranfer rates in the presence of KaiA, $k_{\text{XY}}^{\text{KaiA}}$, depend on how the phosphorylation states change the affinity for KaiA and the energy levels of the monomers, depicted in Fig 3.

#### Ordered phosphorylation of the S and T sites due to phosphotransfer dynamics, nucleotide exchange and differential affinity

It is well known that the S and T sites in each monomer are sequentially phosphorylated through the cycle: U → T → D → S → U [8, 18]. In previous models, this order was imposed by choosing different effective rate constants between the phosphorylation states, in situations of KaiA bound to KaiC, and KaiA not bound to KaiC [8, 9, 24]. However, given the properties of the phosphotransfer reactions and the effects of KaiA as described above, we can now have a more detailed understanding of what makes the phosphorylation cycle go round.

At the start of the phosphorylation phase of the oscillation, the majority of monomers are in the non-phosphorylated state (U), with an ADP in the nucleotide binding pocket and a KaiA bound to the CII domain. The binding of KaiA will enhance the nucleotide exchange rate which increases the fraction of ATP in the binding pocket and consequently forces the phosphotransfer reactions in Eq 1 towards the phosphorylated state while in the mean time blocking the reverse reaction. The next question is why the threonine residue is phosphorylated before the serine residue. Here, differential affinity plays a key role because KaiA binding lowers the free-energy of the T-state and increases that of the S phosphorylation state, as is shown in Fig 3. Together with the fact that the phosphotransfer rate constants of the T site are much faster than those of the S site [8, 29], the threonine residues are phosphorylated before the serine residues. Differential affinity thus has two important effects: Not only does it help to synchronize the KaiC hexamers as found in [9], but it also enforces the correct order of phosphorylation. Since a T-phosphorylated hexamer will still have a high affinity for KaiA, the ATP fraction in the binding pockets will remain high such that eventually both the serine and threonine sites are phosphorylated and the monomers arrive in the D state.

In the dephosphorylation phase of the oscillation, when all KaiA is sequestered and therefore no nucleotide exchange is possible in the CII domain, the ATP in the CII binding pockets will eventually be hydrolyzed. With ADP in the binding pocket, phosphotransfer reactions can occur causing dephosphorylation [28]. Without KaiA bound to CII, the serine residue becomes energetically favorable over the threonine residue again and because phosphotransfer with the threonine residue is faster than with the serine residue, (meaning that the D ⇌ S transitions are faster than the D ⇌ T transitions) the majority of the D phosphorylated monomers will proceed to the S state instead of the T state. The S-site will slowly further dephosphorylate to the U state. This shows how differential affinity and nucleotide exchange together give rise to the ordered phosphorylation of the monomers.

#### ADP in solution slows down phosphorylation

Experiments show that the fraction of ATP in solution, *α*_ATP_ = [ATP]/([ATP]+[ADP]), has a significant effect on the phosphorylation speed and the amplitude of the oscillations in the in-vitro system [27, 41, 53]. They also show that this sensitivity to the ATP fraction is the primary input for entraining the oscillator to the daily day-night cycle [27, 41, 53]. As explained above, our model exhibits this sensitivity because the binding probabilities for ATP and ADP to the binding pocket of the CII domain are directly proportional to the ATP and ADP fraction, respectively, as given in Eqs 2 and 3.

### Model of the KaiC power cycle

In the previus section we discussed the phosphorylation dynamics of KaiC and the interaction between its CII domain and KaiA, and how these effects combine to generate the ordered phosphorylation of the threonine and serine sites in KaiC. The CI domain does not seem to play a crucial role here, in particular since the phosphorylation dynamics in the presence of KaiA only, is unaffected in a KaiC mutant where hydrolysis in the CI domain is deactivated [27]. This raises the question of what role the CI domain fulfills in the Kai oscillator. Here we describe how hydrolysis in CI together with the binding of KaiB to the CI domain drives the conformational switch of the hexamer and how the slow binding of KaiB, together with the subsequent sequestration of KaiA, helps to synchronize the ensemble of KaiC hexamers.

#### Nucleotide exchange in and KaiB binding to the CI domain drives the conformational switch of KaiC

*KaiB and ADP binding to CI is cooperative*. Experiments show that the binding of KaiB requires catalytic activity of the CI domain, since a mutant that lacks the hydrolysis site does not bind KaiB [27]. Furthermore, when KaiB is added to a solution with only KaiC and ATP, the ATPase rate drops significantly [25]. Because KaiA is not present in both experiments, the ATPase activity in the CII domain is negligible as explained in the section on KaiA acting as a nucleotide exchange factor, such that the change in the ATPase rate can be attributed to changes in the CI domain. Given these results, it seems likely that the affinity of KaiB for KaiC depends on ADP in the CI binding pockets created by ATP hydrolysis, and that, vice versa, KaiB binding stabilizes the binding of ADP [38].

In our model, the conformational switch from the active to inactive state depends on ATP hydrolysis in the CI domain and the binding of KaiB to the CI domain. Specifically, both KaiB and ADP binding to CI stabilize the inactive conformational state. It is a characteristic of the MWC model that this introduces an effective cooperativity between KaiB and ADP: KaiB binding enhances the probability that KaiC is in the inactive state, in which ADP will then remain bound more strongly; conversely, ADP in CI will increase the likelihood that KaiC is in the inactive state, in which it will bind KaiB more strongly.

*KaiB and ADP binding to CI stabilize the inactive conformational state*. For simplicity, there is no direct cooperativity between ADP and KaiB binding, such that the free-energy difference between the active and inactive conformation of the hexamer is proportional to the number of ADP nucleotides, *n*^CI·ADP^, and KaiB monomers, *n*^CI·KaiB^,

$$\begin{array}{c} \Delta G_{A,I}^{\text{hex}}=\left(n_{}^{\text{CI}\cdot \text{ADP}}-n_{0}^{\text{CI}\cdot \text{ADP}}\right)\;\delta g_{A,I}^{\text{ATP},\text{ADP}}+n_{}^{\text{CI}\cdot \text{KaiB}}\;\delta g_{A,I}^{\text{CI}\cdot \text{KaiB}}. \end{array}$$ (10)

Here, $\delta g_{A,I}^{\text{ATP},\text{ADP}}=\delta g_{I}^{\text{ATP},\text{ADP}}-\delta g_{A}^{\text{ATP},\text{ADP}}$, is the difference in the free-energy increase upon converting one ATP into an ADP in the CI domain, between the inactive and active conformational state of the hexamer. Since the experiments indicate that the stability of the inactive state increases with the number of bound ADP molecules as discussed above, ADP needs to have a higher affinity for the CI domain in the inactive state as compared to the active state. On the other hand, the exchange of ADP for ATP should be energetically favorable, $\delta g_{I}^{\text{ATP},\text{ADP}}>0$, such that ADP can be exchanged spontaneously at the end of the phosphorylation cycle. These two conditions can be satisfied by choosing $\delta g_{A}^{\text{ATP},\text{ADP}}>\delta g_{I}^{\text{ATP},\text{ADP}}>0$ such that $\delta g_{A,I}^{\text{ATP},\text{ADP}}<0$. These conditions indeed ensure that ATP hydrolysis stabilizes the inactive state, while still allowing for spontaneous ADP release at the end of the cycle. The free-energy difference between the active and inactive state when all CI binding pockets have ATP bound, is set in Eq 10 via the parameter $n_{0}^{\text{CI}\cdot \text{ADP}}$, and determines the threshold number of bound ADP molecules that are required to make the inactive state more stable that the active one.

Finally, the free-energy contribution to $\Delta G_{A,I}^{\text{hex}}$, from binding a single KaiB monomer follows from the dissociation constants for KaiB binding to the active and inactive state, $K_{d,A}^{\text{CI}\cdot \text{KaiB}}$ and $K_{d,I}^{\text{CI}\cdot \text{KaiB}}$, respectively, via the detailed balance relation $\delta g_{A,I}^{\text{CI}\cdot \text{KaiB}}=-\log(K_{d,A}^{\text{CI}\cdot \text{KaiB}}/K_{d,I}^{\text{CI}\cdot \text{KaiB}})$. A higher affinity of KaiB for inactive KaiC than for active KaiC, $K_{d,A}^{\text{CI}\cdot \text{KaiB}}/K_{d,I}^{\text{CI}\cdot \text{KaiB}}>1$, means that KaiB binding stabilizes the inactive state, $\delta g_{A,I}^{\text{CI}\cdot \text{KaiB}}<0$. Eq 10 thus shows how ADP and KaiB binding stabilize the inactive conformational state of KaiC.

#### Timing of the conformational switch is determined by phosphorylation of CII domain, which sets ADP dissociation rate in CI domain

The switch from the active to the inactive state is driven energetically by hydrolysis of ATP in the CI domain and the subsequent binding of KaiB. But what exactly sets the timing of this switch?

*Phosphorylation of the CII domain controls ADP dissociation rate in CI domain*. Interestingly, experiments show that the binding of KaiB requires not only the hydrolysis of ATP in CI, but also that the CII domain is phosphorylated at least on the serine residue [27]. The latter observation might be the result of a direct interaction of KaiB with the CII domain, but could also be due to an indirect effect, in which the likelihood that CI is bound to ADP (which enhances KaiB binding), depends on the phosphorylation state of CII. The latter hypothesis is supported by the experimental observation that hyperphosphorylated KaiC has a lower ATPase activity and a higher fraction of ADP in the binding pockets, as compared to non-phosphorylated KaiC [25, 26, 39]. As mentioned before, the lower measured ATPase activity in hyperphosphorylated KaiC must, because of the absence of KaiA in these experiments, be attributed to the CI domain, and not to changes in the CII domain. The lower ATPase rate is the result of a lower hydrolysis rate and/or a lower ADP dissociation rate. However, a lower hydrolysis rate with a constant ADP dissociation rate would lead to a lower ADP fraction in the binding pockets, in contrast to what has been observed experimentally [28, 39]. We thus conclude that the phosphorylation state of CII determines the ATPase rate of CI through the ADP dissociation rate: As serine residues on CII become phosphorylated, the ADP dissociation rate at the CI domain decreases. These arguments indicate that the regulatory mechanism that controls the timing of the conformational switch is the dependence of the dissociation rate of ADP in the CI domain on the phosphorylation state of the CII domain.

**Fig 4:**
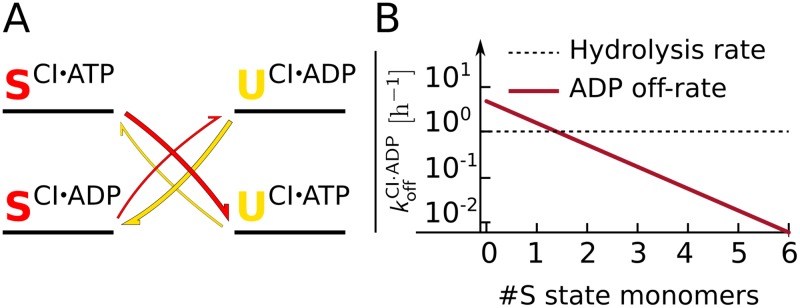
The phosphorylation state of the CII domain regulates the ADP fraction in the CI domain by changing the ADP release rate, but not its affinity. (A) Cartoon that illustrates why the experiments rule out the latter “affinity” scenario. The cartoon shows a free-energy landscape for this affinity scenario in which the phosphorylation state of CII determines the affinity of ADP for CI; specifically, the cartoon illustrates the case where a monomer in the S state stabilizes ADP in the binding pocket of the CI domain. The superscripts denote the state of the CI binding pocket. The S-phosphorylated state lowers the free energy of the CI⋅ADP state compared to the U state, thereby decreasing the ADP-off rate in the CI binding pocket. It follows from detailed balance that when the CI domain has ADP bound, the phosphotransfer from S to U is uphill in free energy, which decreases the phosphotransfer rate. The diagonal arrows give the phosphotransfer reactions from U to S (yellow) and S to U (red). When the CI domain is predominantly in the ATP state, as was done in [27] by lowering the hydrolysis rate with a mutation, the affinity scenario, as illustrated in the cartoon, would predict that the dephosphorylation rate from S to U increases. However, the experiments show no significant change in phosphotransfer rates, making this affinity scenario unlikely. Based on these arguments, we predict that phoshorylation of CII does not change the affinity, but does change the absolutes rates of ADP dissociation and association. (B) ADP dissociation rate as a function of the number of S state monomers in the hexamer, assuming the other monomers are in the U state. Between 1 and 2 monomers in the S state, the dissociation rate is higher than the hydrolysis rate which puts the binding pockets of CI domain predominantly in the ATP state, driving the hexamer to the active conformational state.

*The ADP-CI association and dissociation rates change, but their ratio, the affinity, does not*. A question not yet answered is whether the phosphorylation of the CII domain changes the magnitude of the ADP binding rates, or also the affinity, which depends on the ratio of the association and dissociation rates. Recent experiments allow us to answer this question. These experiments show that both the phosphorylation and dephosphorylation rates are unchanged in a KaiC mutant where ATP hydrolysis in the CI domain is deactivated, which decreases the fraction of bound ADP [27]. Detailed balance would entail that if phosphorylation of CII were to stabilize ADP in CI, then vice versa ADP would stabilize the phosphorylated state; KaiC hexamers with fewer ADP molecules in CI, such as the KaiC mutant in [27], would then dephosphorylate faster. Fig 4A illustrates how the detailed balance condition changes the phosphotranfer rates in this case. Since the experiments show that the (de)phosphorylation rates are unchanged [27], we must conclude that the affinity of ADP for CI does not depend on the phosphorylation state of the CII domain. Hence not only the dissociation rate of ADP changes, but also the association rate changes by the same factor, leaving the affinity unchanged.

*Phosphorylation of CII controls ADP dissociation from CI via transition state*. To explain the dependence of the ADP fraction in the CI domain on the phosphorylation state of the CII domain, we envision a model in which the phosphorylation state of CII affects a short-lived transition state for the dissociation of ADP from the nucleotide binding pocket of CI. In our model, the activation energy for ADP dissociation in each monomer, $\Delta G_{\text{act}}^{\text{CI}\cdot \text{ADP}}$, depends linearly on the phosphorylation state *X*_*i*_ of all monomers in the hexamer

$$\begin{array}{c} \Delta G_{\text{act}}^{\text{CI}\cdot \text{ADP}}=\sum_{i=1}^{6}\delta g_{\text{act},A/I}^{\text{CI}\cdot \text{ADP}}\left(X_{i}\right), \end{array}$$ (11)

where $\delta g_{\text{act},A/I}^{\text{CI}\cdot \text{ADP}}$ is the contribution of a single monomer on the activation energy in the active (A) or inactive (I) conformational state. The ADP dissociation rate is then given by

$$\begin{array}{c} k_{\text{off}}^{\text{CI}\cdot \text{ADP}}=k_{\text{off},0}^{\text{CI}\cdot \text{ADP}}\;\exp\left(-\Delta G_{\text{act}}^{\text{CI}\cdot \text{ADP}}\right), \end{array}$$ (12)

where $k_{\text{off},0}^{\text{CI}\cdot \text{ADP}}$ is the off-rate when the hexamer is in the active state with all monomers in the U-state.

Fig 4B shows the ADP dissociation rate as a function of the number of monomers in the S state for a hexamer in the inactive conformation, assuming that the other monomers are in the U state, which is typically the case during the dephosphorylation phase of the cycle. The energy values $\delta g_{\text{act},I}^{\text{CI}\cdot \text{ADP}}$(X) determine when the rate of ADP dissociation (typically leading to ATP binding) will be higher than the ATP hydrolysis rate (leading to the ADP bound state). We choose the energy values such that the crossover in the rates happens between 1 and 2 monomers in the S-state. Hence, when a hexamer has fewer than 2 monomers in the S-state, ADP will be released and ATP becomes bound, and the hexamer will switch back to the active conformation, completing the cycle.

*ADP-CI association rate is very low and ADP arises only via hydrolysis of bound ATP*. Because ADP in the CI binding pocket is energetically very unfavorable [30–32], and the affinity of KaiC for KaiB does not seem to depend on the bulk ATP fraction [27, 53], the association rate of ADP from the bulk to the CI binding pocket will be very low. Therefore, in our model, regardless of the bulk ATP fraction, ADP can only appear in the CI binding pocket through the hydrolysis of ATP:

$$\begin{array}{c} k_{\text{ATP}\to \text{ADP}}^{\text{CI}}=k_{\text{hyd}}^{\text{CI}}. \end{array}$$ (13)

Moreover, since the ADP association rate is assumed to be zero, after ADP dissociation the pocket will always bind ATP. Since ATP association is much faster than nucleotide dissociation [39], as exploited in the section on phosphorylation dynamics, the rate of exchanging ADP for ATP is simply given by the ADP dissociation rate:

$$\begin{array}{c} k_{\text{ADP}\to \text{ATP}}^{\text{CI}}=k_{\text{off}}^{\text{CI}\cdot \text{ADP}}, \end{array}$$ (14)

with the latter given by Eq 12.

#### T and S phosphorylation states have an antagonistic effect on the ADP fraction in CI

Experiments show that a phosphomimetic of the T state of KaiC has a higher ATPase activity compared to unphosphorylated KaiC and that a phosphomimetic of the S state has a lowered ATPase activity [54]. This is likely the result of an antagonistic effect of phosphorylation of the threonine and serine sites on the dissociation rate of ADP. Specifically, in our model monomers in the T state will lower the activation energy for ADP release, $\delta g_{\text{act}}^{\text{CI}\cdot \text{ADP}}\left(T\right)<0$, while monomers in the S state increase the activation energy, $\delta g_{\text{act}}^{\text{CI}\cdot \text{ADP}}\left(S\right)>0$. Because the S and T sites are orderly phosphorylated, their antagonistic effect on the dissociation rate of ADP will create a sharp transition between the phase in which the ADP fraction in CI is low and that in which it it is high [24]. Furthermore, just like in the push-pull network studied by Goldbeter *et al*. [55], the ATP fraction in the CI domain will depend on the *difference* between the monomers in the S and T state, and not on their absolute number. This makes the regulation of the CI domain less sensitive to the absolute phosphorylation level, which depends on the bulk ATP fraction [27, 53].

#### Nucleotides, KaiB and KaiA binding to the CI domain stabilizes the inactive state

Detailed balance implies that the different affinities of the binding partners for the active and inactive state of KaiC, is reflected in the free-energy difference between the two conformations, $\Delta G_{A,I}^{\text{hex}}$. In our model, we assume there is no cooperative binding to KaiC in a given conformational state (although the MWC model introduces an effective cooperativity as explained in the theory section on the power cycle), such that we can split the dependence on each binding partner in independent terms,

ΔGA,Ihex=nCI·ADP-n0CI·ADPδgA,IATP,ADP+nCI·KaiBδgA,ICI·KaiB=+nCI·KaiAδgA,ICI·KaiA+nCII.KaiAδgA,ICII·KaiA. (15)

Each contribution is directly proportional to the the number of ADP nucleotides, KaiB monomers and KaiA dimers, *n*^CI·ADP^, *n*^CI·KaiA^, and *n*^CII·KaiA^, respectively, bound to the CI domain. The last contribution is proportional to the number of KaiA dimers, *n*^CII·KaiA^, bound to the CII domain. The terms depending on the number of ADP and KaiB proteins bound are explained in the theory section. As discussed in more detail in the next section, KaiA can only bind to the CI domain when 6 KaiB monomers are bound to CI; moreover, 6 KaiA dimers can then be sequestered. The free-energy contribution for binding a single KaiA dimer to CI results from the detailed-balance relation, $\delta g_{A,I}^{\text{CI}\cdot \text{KaiA}}=-\log(K_{d,A}^{\text{CI}\cdot \text{KaiA}}/K_{d,I}^{\text{CI}\cdot \text{KaiA}})$, where $K_{d,A}^{\text{CI}\cdot \text{KaiA}}$ and $K_{d,I}^{\text{CI}\cdot \text{KaiA}}$ are the dissociation constants for the active and inactive state of KaiC, respectively. A similar relation holds for KaiA binding to CII.

**Fig 5:**
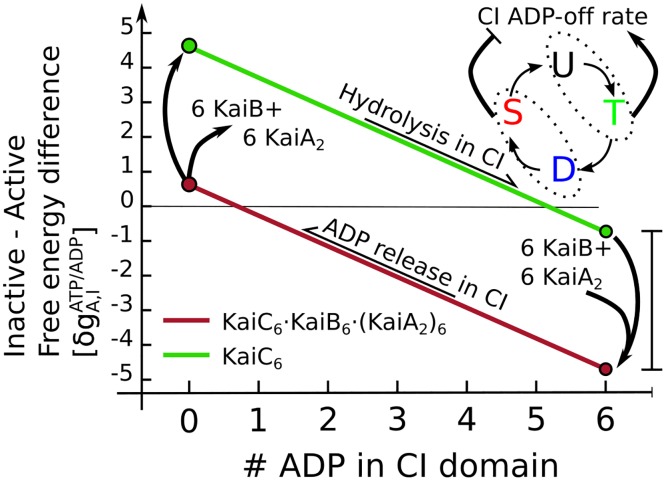
Hydrolysis of ATP in the CI domain, and the subsequent binding of KaiB and KaiA, forms a hysteresis loop in the free-energy difference between the active and inactive state. Starting in the active state with no ADP bound to CI, the free-energy difference linearly decreases as ATP is hydrolyzed to ADP, as indicated by the green line. The number of ADP in CI is set by the competition between a fixed hydrolysis rate and a variable ADP off-rate, which is set by the phosphorylation state of the CII domains of the whole hexamer. Phosphorylation of the T site initially enhances the ADP off-rate, but the subsequent phosphorylation of the S-site will decrease the dissociation of ADP. The antagonistic effect of the cyclically phosphorylated T and S sites on the ADP dissociation rate causes a sharp transition in the CI binding pocket from ATP dominated to ADP dominated [24]. When there are 5 or 6 ADP nucleotides in the CI domain, the hexamer will flip to the inactive state, increasing the affinity for KaiB, which will then slowly bind inactive KaiC. When 6 KaiB monomers are bound to the hexamer, up to six free KaiA dimers will be sequestered from solution. This complex of KaiB and KaiA on the CI domain stabilizes the inactive state of KaiC further (red line). When all KaiA is sequestered, the dephosphorylation of the S-site in the CII domain will increase the nucleotide exchange rate in the CI domain, decreasing the ADP level and increasing the free-energy difference between the active and inactive state. This allows the hexamer to flip back to the active state. In the active state, KaiA and KaiB dissociate from KaiC, because they have a low affinity for CI in the active conformation.

Because KaiA and KaiB have a higher affinity for inactive KaiC than for active KaiC, their binding to the CI domain will stabilize the inactive state. This, together with CI-ATP stabilizing the active state and CI-ADP stabilizing the inactive one, creates a hysteresis loop, as illustrated in Fig 5. Importantly, while KaiA and KaiB binding to CI stabilizes the inactive state with respect to the active one, the system is designed such that when no ADP is bound to CI, the active conformation with KaiA and KaiB bound is *more* stable than the inactive one with KaiA and KaiB bound to it. This is critical, because it ensures that when, during the dephosphorylation phase, the system eventually reaches the point where all ADP has been released and the number of CI-bound ADP molecules has reached zero, the hexamer flips back from the inactive to the active state. In this active state, KaiA and KaiB will then spontaneously dissociate from KaiC, because this conformational state has a low affinity for KaiA and KaiB. Given Eq 15, the requirement that the active state with KaiA and KaiB bound is always more stable than the inactive one when no ADP is bound to CI, entails that $n_{0}^{\text{CI}\cdot \text{ADP}}\;\delta g_{A,I}^{\text{ATP},\text{ADP}}\leq n_{\text{max}}^{\text{CI}\cdot \text{KaiA}}\;\delta g_{A,I}^{\text{CI}\cdot \text{KaiA}}+n_{\text{max}}^{\text{CI}\cdot \text{KaiB}}\;\delta g_{A,I}^{\text{CI}\cdot \text{KaiB}}$.

Given $\Delta G_{A,I}^{\text{hex}}$, the rates of switching from the active to the inactive state, $k_{f}^{\text{conf}}$, and vice versa, $k_{b}^{\text{conf}}$, become $k_{f}^{\text{conf}}=k_{0}^{\text{conf}}\text{exp}(-\Delta G_{A,I}^{\text{hex}}/2)$ and $k_{b}^{\text{conf}}=k_{0}^{\text{conf}}\text{exp}(\Delta G_{A,I}^{\text{hex}}/2)$, respectively. The prefactor $k_{0}^{\text{conf}}$ sets the timescale of switching.

#### Slow KaiB binding sets a time delay between the phosphorylation and dephosphorylation phase

Experiments show the binding of KaiB to the KaiC hexamer is slow due to a combination of a slow conformational switch of KaiB monomers in solution, and a slow reaction step attributed to the CI domain of KaiC [27, 40]. Theoretical modeling suggests that the Kai oscillator requires a time delay between the phase of phosphorylation and the phase of KaiA sequestration and dephosphorylation, in order to generate stable oscillations [9, 16, 24] and a period that is fairly insensitive to changes in the bulk ATP fraction [27]. We introduce this delay by assuming KaiC can not sequester KaiA before a full ring of 6 KaiB monomers has formed on the CI domain [38, 56], and that the rate of KaiB binding to KaiC is slow and independent of the number of KaiB proteins already bound. In this way we simulate the slow appearance of KaiB monomers in the bulk that have a binding competent conformation [40]. As explained in the model overview, we do not explicitly keep track of KaiB, but coarse grain the KaiB concentration in the association rates motivated by the observation that the absolute concentration of KaiB has little influence on the amplitude and period of the oscillation [9, 45].

After a KaiB ring has formed, KaiA will immediately be sequestered from solution due to a very high on-rate, with a maximum of 6 KaiA dimers per hexamer [49, 50]. The affinity of KaiA for the hexamer with a KaiB ring depends on the conformational state of the hexamer: Only in the inactive state KaiA stays bound to KaiC-KaiB. In the active state of KaiC, the CI domain has a lower affinity for both KaiA and KaiB, as discussed in the previous section. Hence, after KaiC has flipped to the active state, KaiA and KaiB will be released, and the cycle starts over.

#### Summary of the cycle dynamics

Hydrolysis of ATP in the CI domain drives the conformational transition from the active state to the inactive one, because ADP in the CI domain stabilizes the inactive state with respect to the active one, see Fig 5. This allows the ensemble of KaiC hexamers to switch from a phosphorylation phase with free KaiA in solution to a dephosphorylation phase where all KaiA is sequestered by KaiC. In the inactive state, the CI domain of KaiC has a high affinity for both KaiA and KaiB, meaning that the complex KaiC_6_ · KaiB_6_ · KaiA_2_)_6_, is energetically very stable.

While all KaiA is sequestered, the KaiC ensemble will dephosphorylate leaving most of the monomers in the U or S state. When the number of S phosphorylated sites drops below a threshold, the energy barrier for ADP release from CI will become sufficiently small, such that ADP will dissociate even though ADP is stabilized by the binding of KaiA and KaiB. Without ADP bound to the CI domain the hexamer returns to the active state, which has a lower affinity for KaiA and KaiB. The sequestered proteins are then immediately released such that the cycle can start over again with an ensemble of hexamers with monomers in the U state.

## Results

### Results on phosphorylation dynamics

To test the validity of our model of the CII domain and to find the correct parameter values shown in Table 2, we compare with the rich body of quantitative experimental results on the in-vitro Kai system. As is done in these experiments, we will study the behavior of different combinations of the main actors: KaiA, KaiB and KaiC and the ATP fraction, *α*_ATP_. First, we will study the dephosphorylation dynamics of phosphorylated KaiC in the absence of KaiA and KaiB. We will investigate the dependence on the ATP fraction and compare to experimental results. Furthermore, we test our hypothesis that phosphotransfer from the threonine and serine sites to ADP is the only possible pathway for dephosphorylation, by comparing to a model where release of the phosphate into the bulk is possible. Next, we study the effects of KaiA on the phosphorylation dynamics and the influence of the bulk ATP fraction on the speed and steady state level of phosphorylation. Lastly, we study if the ordered phosphorylation dynamics of the serine (S) and threonine (T) residues persists when the system of KaiA and KaiC has reached steady state. In the subsequent section, we address the role of KaiB and the oscillatory dynamics. All simulations were performed at the experimental standard concentrations of 0.6μM for KaiA and KaiC, which corresponds to simulating 720 KaiC hexamers and 720 KaiA dimers in a volume of 2 cubic micron.

**Table 2:** Model parameters relating to the CII domain are introduced in the theory section on phosphorylation dynamics and their values are motivated in the results section. Energies are given in units of kT, were k is Boltzmann’s constant and T the temperature.

Parameters relating to the CII domain		
Parameter	Value	Explanation
**Phosphotransfer**		
$k_{\text{UT}}^{0}$	0.50 h^−1^	U⋅ATP→T⋅ADP
$k_{\text{TU}}^{0}$	1.78 h^−1^	T⋅ADP→U⋅ATP
$k_{\text{TD}}^{0}$	0.40 h^−1^	T⋅ATP→D⋅ADP
$k_{\text{DT}}^{0}$	0.20 h^−1^	D⋅ADP→T⋅ATP
$k_{\text{SD}}^{0}$	1.50 h^−1^	S⋅ATP→D⋅ADP
$k_{\text{DS}}^{0}$	2.00 h^−1^	D⋅ADP→S⋅ATP
$k_{\text{US}}^{0}$	0.15 h^−1^	U⋅ATP→S⋅ADP
$k_{\text{SU}}^{0}$	0.20 h^−1^	S⋅ADP→U⋅ATP
**Nucleotide binding pocket**		
$k_{\text{hyd}}^{\text{CII}}$	1.00 h^−1^	ATP hydrolysis rate
$k_{\text{off},\text{KaiA}}^{\text{CII}\cdot \text{ADP}}$	6.00 h^−1^	ADP off-rate with KaiA bound
$K_{\text{ATP}/\text{ADP}}^{\text{CII}}$	0.10	Relative affinity for ATP and ADP
**KaiA affinity**		
$k_{\text{on},0}^{\text{CII}\cdot \text{KaiA}}$	-1.00 mM h^−1^	KaiA on-rate for CII domain
$k_{\text{off},0}^{\text{CII}\cdot \text{KaiA}}$	-1.00 h^−1^	KaiA off-rate for CII domain
$\delta g_{\text{bind}}^{\text{CII}\cdot \text{KaiA}}\left(U\right)$	0.00 kT	Free energy effect of U-monomer
$\delta g_{\text{bind}}^{\text{CII}\cdot \text{KaiA}}\left(T\right)$	-0.30 kT	Free energy effect of T-monomer
$\delta g_{\text{bind}}^{\text{CII}\cdot \text{KaiA}}\left(D\right)$	1.00 kT	Free energy effect of D-monomer
$\delta g_{\text{bind}}^{\text{CII}\cdot \text{KaiA}}\left(S\right)$	2.00 kT	Free energy effect of S-monomer
$\Delta G_{A,I}^{\text{CII}\cdot \text{KaiA}}$	10 kT	Free energy effect of conformation

#### KaiC dephosphorylation speed is set by phosphotransfer rates

**Fig 6:**
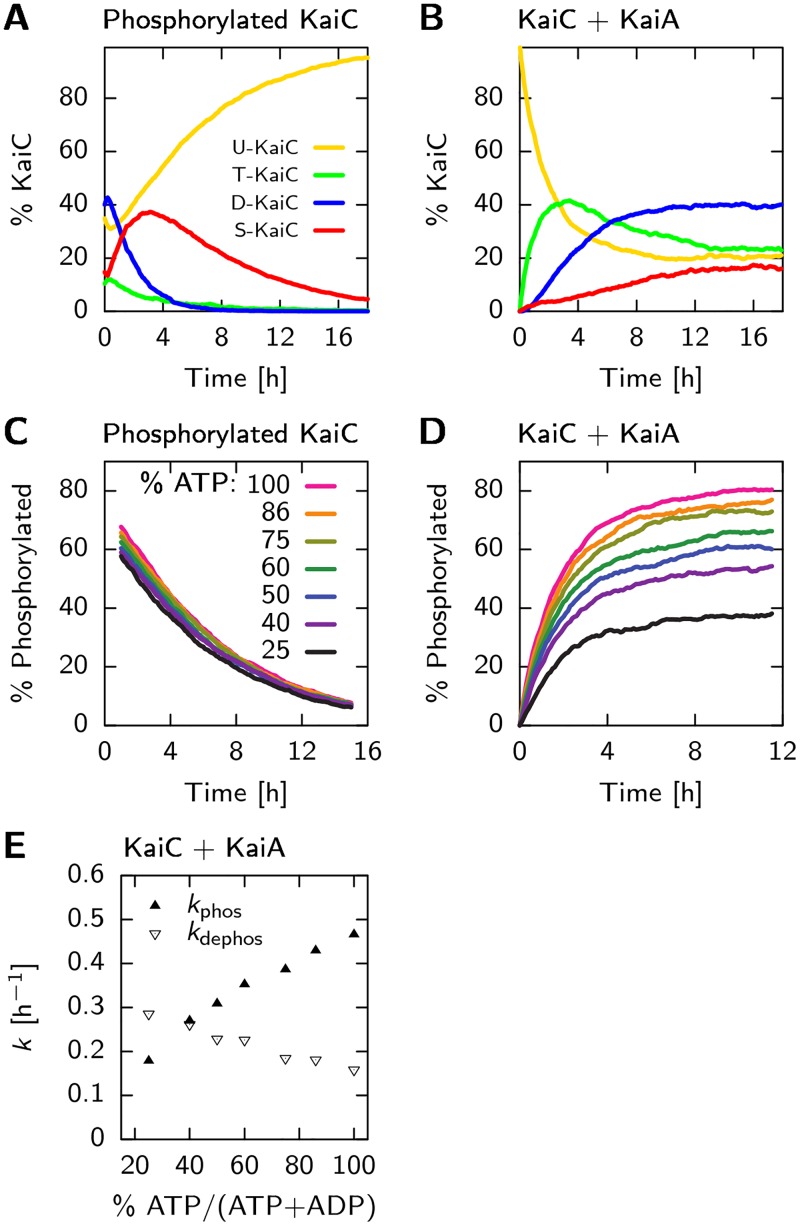
The free-energy landscape in Fig 3, accurately describes the phosphorylation and dephosphorylation dynamics in the CII domain, both with and without KaiA and for a wide range of different ATP to ADP fractions in the solution, *α*_ATP_. (A) Dephosphorylation dynamics, starting with phosphorylated KaiC and (B) phosphorylation dynamics, starting with unphosphorylated KaiC and KaiA. (C) Dephosphorylation and (D) phosphorylation of KaiC under similar conditions as in panels A and B, respectively, but now for different bulk fractions of ATP, *α*_ATP_. Consistent with experiments, the rate of dephosphorylation is independent of *α*_ATP_, but the phosphorylation-rate does depend strongly on this fraction. (E) Rates of (de)phosphorylation in panel D, found by fitting Eq 16 to the first four hours of phosphorylation data.

*Dephosphorylation via phosphotransfer can reproduce experiments*. We start with simulating an ensemble of KaiC hexamers in a 100% ATP solution, and compare with the experimental results presented in the supplementary information of [27]. Initially, KaiC is highly phosphorylated. As there is no KaiA (and also no KaiB), this experiment allows us to distinguish the rate constants related to phosphotransfer dynamics given in Eq 1, from the effects related to the interaction with KaiA. To obtain the rapid decay of the T and D phosphorylated states and the transient peak in the S state, as shown in experiments, the phosphotransfer rates relating to the threonine site have to be significantly faster than the rates relating to the serine site. Furthermore, dephosphorylation is downhill in free energy. Otherwise, the ATP regenerated in the dephosphorylation reaction would phosphorylate KaiC again. The free-energy landscape of the phosphorylation states combined with the nucleotide binding pockets, where KaiA is not bound, is drawn in Fig 3, left panel. We choose the magnitude of the rates such that we can reproduce the time and height of the maximum in the concentration of S-phosphorylated KaiC as well as its subsequent decay. Since dephosphorylation can only occur after the ATP in the binding pocket has been hydrolyzed, the hydrolysis rate constant sets an upper bound on the speed, which we set to $k_{\text{hyd}}^{\text{CII}}=1{\text{ h}}^{-1}$, similar to what has been found in [28]. Fig 6A shows that our model, with the phosphotranfer parameters of Table 2, reproduces the dephosphorylation dynamics of Fig. S2 of [27]. Next, we test if in our model the dephosphorylation speed is independent of the bulk ATP fraction, *α*_ATP_ = [ATP]/([ATP] + [ADP]), as was found in experiments [53]. Eq 1C shows that this is indeed the case. This is because the ATP hydrolysis rate is higher than the ATP dissociation rate and the phosphotransfer rate is faster than the ADP dissociation rate. This ensures that during dephosphorylation the nucleotides are not released—if this would happen, the subsequent nucleotide binding and hence the dephosphorylation rate would depend on *α*_ATP_. When the above requirements are fulfilled, KaiC predominantly dephosphorylates via $\text{D}\cdot \text{ATP}\overset{k_{\text{hyd}}^{\text{CII}}}{\to }\text{D}\cdot \text{ADP}\overset{k_{\text{DS}}^{0}}{\to }\text{S}\cdot \text{ATP}\overset{k_{\text{hyd}}^{\text{CII}}}{\to }\text{S}\cdot \text{ADP}\overset{k_{\text{SU}}^{0}}{\to }\text{U}\cdot \text{ATP}$.

**Fig 7:**
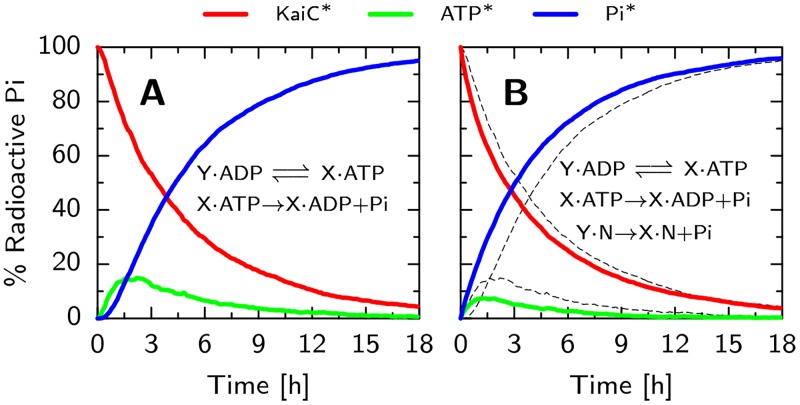
Phosphotransfer between KaiC and ADP is the dominant pathway during dephosphorylation of KaiC. Initial conditions are the same as in Fig 6A, but now the phosphate groups are ‘radioactive’ such that they can be tracked from their initial position on the KaiC (KaiC*, red line), to the radioactive ATP (ATP*, green line) and in solution (Pi*, blue line). Panels A show time traces of this dephosphorylation assay for our model, while panel B shows the results for an alternative model in which spontaneous dephosphorylation is also possible (for comparison, the results of our model, presented in panel A, are shown as thin dashed lines). In our model, panel A, dephosphorylation can only occur via the transfer of the phosphate group on KaiC to the ADP in the CII nucleotide binding pocket. The alternative model, panel B, represents a scenario with two dephosphorylation pathways: one via the transfer to ADP, and one via direct exchange with the bulk. We chose rates such that dephosphorylation occurs equally through both pathways, and the total dephosphorylation speed is similar to our model (panel A). Comparing with the radioactive phosphate tracking experiment in [28], Fig 2, shows that the scenario where dephosphorylation can only occur via ADP (panel A) agrees best with the data. In particular, the onset of the free Pi concentration shows a temporal delay in panel A that is absent in panel B. Furthermore, the level of radioactive ATP is the highest in panel A, which compares best to the 20% peak height in [28].

*Dephosphorylation does not occur via phosphate release*. Importantly, if dephosphorylation were to occur through the direct release of inorganic phosphate groups into the bulk, then the dephosphorylation speed would *also* trivially be independent of the bulk ATP fraction. We therefore set out to test the hypothesis that all phosphorylation and dephosphorylation occurs only through phosphotransfer [28, 29]. To this end, we compare our model with an alternative one in which also direct exchange of the phosphate group with the bulk is possible. Specifically, we simulated the experiments performed in [28], in which they track radioactively labeled phosphate groups starting bound to the serine and threonine sites of KaiC, in a solution with only non-radioactive ATP. Here, KaiC dephosphorylates while producing a transient population of radioactive ATP*, where at the maximum around 20% of the radioactive phosphates are bound to a nucleotide. The inorganic phosphate groups, Pi*, only appear in the bulk after a marked delay. Fig 7A shows that our model, where dephosphorylation can only occur via phosphotransfer of the phosphate to the ADP and the subsequent hydrolysis of the ATP, is in good quantitative agreement with the results of Fig. 2 in [28]: Both the magnitude and timing of the peak in the radioactive ATP, ATP*, and the delay in the appearance of radioactive phosphate groups in the bulk, Pi*, are in good agreement.

To test the effects of direct exchange of phosphate with the bulk, we add the reaction, X · N ⇌ Y · N + Pi, to our model. Here X and Y are connected phosphorylation states and N is the state of the nucleotide binding pocket, which can be either ATP or ADP, but now does not change state during dephosphorylation. We changed the rate constants such that dephosphorylation occurs equally through both pathways and the overall speed is comparable to the original model. Fig 7B shows that in this scenario, the time traces are qualitatively different from the experiment. Because the phosphate groups are directly exchanged with the bulk, the delay in [Pi*] has disappeared and the magnitude of the peak in radioactive ATP is less than 10%. The discrepancy between the experimental data and our simulations thus confirms our hypothesis that the direct exchange with the bulk of phosphates is negligible.

#### KaiA sets phosphorylation dynamics

To address the effect of KaiA binding to the CII domain on the phosphorylation free-energy landscape shown in Eq 4, we compared with the experimental time traces in Fig. S2 of [27]. In these experiments, they start with unphosphorylated KaiC together with KaiA and track the fractions of T,D and S phosphorylated KaiC. This allows us to constrain the change in free energy of the phosphorylation states due to KaiA binding, $\delta g_{\text{bind}}^{\text{CII}\cdot \text{KaiA}}\left(X\right)$, introduced in Eq 4. The affinity of KaiA for unphosphorylated KaiC is about 1nM, as reported in [50]. To make the large overshoot of T-phosphorylated KaiC possible, KaiA should lower the free-energy of the T state, $\delta g_{\text{bind}}^{\text{CII}\cdot \text{KaiA}}\left(T\right)<0$, while at the same time blocking the transition to the S-phosphorylation state, $\delta g_{\text{bind}}^{\text{CII}\cdot \text{KaiA}}\left(S\right)>0$. Experiments starting with radioactively labeled nucleotides in the binding pockets of KaiC in solution with KaiA, show that ADP bound to the CII domain has a very high off-rate [39]. Therefore, the ADP dissociation rate with KaiA bound to the CII domain has a high value of $k_{\text{off},\text{KaiA}}^{\text{CII}\cdot \text{ADP}}=6.0{\text{h}}^{-1}$. Taken together, we find that with the parameters for KaiA binding presented in Table 2, we can reproduce the phosphorylation dynamics as shown in Fig 6B.

*Model can reproduce dependence of phosphorylation on ATP fraction*. Next we checked the effect of the ATP fraction, *α*_ATP_, on the speed and steady state level of the total phosphorylation fraction and compare with experiments in [53]. As the sensitivity of phosphorylation to the bulk ATP fraction is set by the relative affinity for ATP and ADP, $K_{\text{ATP}/\text{ADP}}^{\text{CII}}$, we can use the data in [53] to constrain this parameter. Using $K_{\text{ATP}/\text{ADP}}^{\text{CII}}=0.10$, Fig 6D shows that the phosphorylation time traces at different values of *α*_ATP_, are in good agreement with experiments: Changing *α*_ATP_ from 100% to 25%, the steady state phosphorylation level drops from 80% to 40%.

To quantify the change in phosphorylation speed by varying *α*_ATP_, we fit the first 4 hours of the phosphorylation time traces in Fig 6D, *p*(*t*), with a 2 state model,

$$\begin{array}{c} p\left(t\right)=k_{\text{phos}}/\left(k_{\text{phos}}+k_{\text{dephos}}\right)\;(1-\text{exp}\left(-\left(k_{\text{phos}}+k_{\text{dephos}}\right)t\right). \end{array}$$ (16)

Here, the system switches between the phosphorylated and dephosphorylated state with the rates *k*_phos_ and *k*_dephos_. Fig 6E shows that *k*_phos_ linearly increases with *α*_ATP_, as was found in [27, 53], but with a slope that is less steep. Furthermore, fitting the two state model to our modeling data yields a *k*_dephos_ that decreases with *α*_ATP_, while the fitting to the experimental data yields a *k*_dephos_ that is virtually independent of *α*_ATP_. This decrease in our model is due to the fact that the effective dephosphorylation rate is proportional to the fraction of ADP in the CII binding pocket. We attribute the inconsistency, at least in part, to the lower number of data points in the experimental time traces that are available for fitting to the two state model.

#### Ordered phosphorylation of the S and T sites persists in steady state

The phosphotransfer reactions and the binding of KaiA all fulfill detailed balance, and only the irreversible hydrolysis reaction in the CI and CII domains do not. This raises the question whether, when the solution contains only KaiC hexamers and KaiA, individual KaiC monomers continue to go through the ordered cycle U → T → D → S → U. In this case there will be no macroscopic oscillations in the phosphorylation fraction because KaiA is never sequestered by KaiB and the phosphorylation cycles of the individual KaiC hexamers are not synchronized. The concentrations of the U,T,D and S phosphorylated monomers will therefore be in steady state. If we find ordered phosphorylation of the threonine and serine sites, this has to be driven by the hydrolysis of ATP in the CI and/or the CII domain. We want to know whether the phosphorylation cycle is mainly driven by hydrolysis in the CI or the CII domain. To this end, below we consider scenarios where we remove the hydrolysis in the respective domains and study its effect on the phosphorylation dynamics.

To find out if the ordered phosphorylation cycle persist in a system with only KaiA and KaiC, we need to know if there are net fluxes between states in the phosphorylation state space, indicating that detailed balance is broken [57]. To this end, we keep track of the number of phosphorylated threonine, *n*_T_(t), and serine residues, *n*_S_(t), in each individual hexamer in the ensemble. From this data we can calculate the probability, $P_{n_{T}^{},n_{S}^{}}$, that a hexamer is in phosphorylation state (*n*_T_, *n*_S_), and the number of times the hexamer switches from this state to one of its neighboring states, $N_{\alpha ,\beta }^{x}$. Here *x* ∈ {*T*, *S*} indicates whether the (de)phosphorylation event involved a threonine or serine residue, *α* gives phosphorylation state (*n*_T_, *n*_S_) before the transition, and *β* the phosphorylation state after the transition. The net flux between two neighboring states is given by

$$\begin{array}{c} W_{\alpha ,\beta }^{x}=\frac{N_{\alpha ,\beta }^{x}-N_{\beta ,\alpha }^{x}}{\Delta t_{\text{sim}}^{}}, \end{array}$$ (17)

where Δ*t*_sim_ is the time interval over which these time traces were measured. As was done in [58], we can define a vector, ${\vec{J}}_{n_{T}^{},n_{S}^{}}$, that points in the direction of the mean net flux through the state (*n*_T_,*n*_S_)

$$\begin{array}{c} {\vec{J}}_{n_{T}^{},n_{S}^{}}=\frac{1}{2}\left(\begin{array}{c} W_{\alpha^{-},\alpha }^{T}+W_{\alpha ,\alpha^{+}}^{T}\\ W_{\alpha^{-},\alpha }^{S}+W_{\alpha ,\alpha^{+}}^{S} \end{array}\right). \end{array}$$ (18)

Here the pair *α*^−^, *α* indicates the net flux along the threonine or serine axis from below the coordinate *α*, and the pair *α*, *α*^+^ indicates the net flux to above *α*.

Hydrolysis of ATP in the CII domain provides the ADP required for dephosphorylation. However, when there is no hydrolysis in the CII domain and the bulk only contains ATP, ATP is never converted to ADP in the CII domain (See Fig 2), such that dephosphorylation becomes impossible. All the monomers will be permanently in the D state with an ATP in the binding pocket, blocking the possibility of a phosphorylation cycle. Therefore, in all scenarios discussed in this section, we will use a lower ATP fraction of *α*_ATP_ = 0.5 such that there is ADP from the bulk available for dephosphorylation.

**Fig 8:**
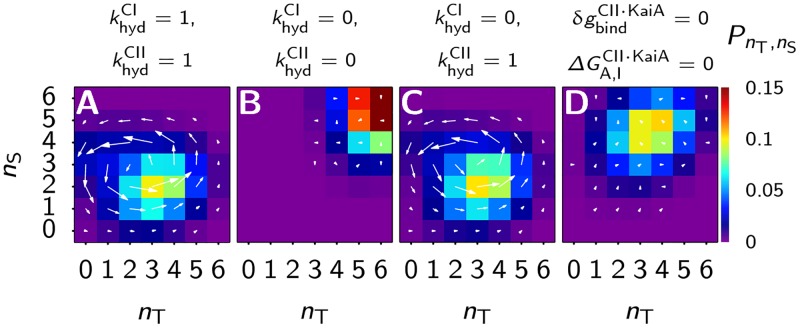
Hydrolysis in the CII domain, without KaiA sequestration by the CI domain, is sufficient to generate the ordered phosphorylation of the T and S sites in a solution of KaiC and KaiA. In all panels, the bulk ATP fraction is 50%. (A-D) Heatmaps of the probability, $P_{n_{T}^{},n_{S}^{}}$, for a single hexamer, of having *n*_T_ phosphorylated threonine sites and *n*_S_ phosphorylated serine sites. Arrows indicate the net flux through a state, where the length is proportional to the magnitude of the flux. (A) KaiC and KaiA, with hydrolysis of ATP in both the CI and CII domains, shows a clear ordered cycle in state space. (B) Without hydrolysis in both the CI and CII domains, all the equations in our model obey detailed balance, and the fluxes in state space disappear. (C) When we remove hydrolysis in the CI domain, the fluxes in phosphorylation state space are little affected compared to panel A. (D) When there is hydrolysis in both domains, but no differential affinity of the CII domain for KaiA, the hydrolysis cycle in the CII domain does not couple to the phosphorylation cycle, and there is no ordered phosphorylation.

In Fig 8, we show a heat map of $P_{n_{T}^{},n_{S}^{}}$ together with the vectors ${\vec{J}}_{n_{T}^{},n_{S}^{}}$, for a system with KaiA and KaiC, in the presence of ATP hydrolysis in both the CI and CII domains. In panel A we show the behavior of KaiC in solution with KaiA, which clearly shows cyclic net fluxes in phosphorylation state space. Starting in the lower left corner, where the hexamer is unphosphorylated, first the threonine sites will phosphorylate after which the serine sites become phosphorylated. After reaching the upper right corner of the state space, the hexamer will first dephosphorylate the threonine sites and then the serine sites. As explained in the theory section on phosphorylation dynamics, the combination of hydrolysis in the CII domain and differential affinity for KaiA, results in a high ATP fraction in the CII binding pockets when the hexamer is predominantly in the U and T state, and a low ATP fraction when it is in the D and S state. Therefore, KaiC, on average, phosphorylates when it is in the U and T state and dephosphorylates when it is in the D and S state, which is the origin of the cycle in *n*_T_ − *n*_S_ space.

*Cycle is driven by ATP hydrolysis and differential affinity*. We then asked what drives the phosphorylation cycle at the level of the individual hexamers. We first set the hydrolysis rates in both domains to zero, thereby removing all irreversible pathways in the model. Clearly, without ATP hydrolysis, as shown in Fig 8B, the fluxes disappear completely. This shows that our model obeys detailed balance when we remove the two irreversible pathways. When we remove hydrolysis in CI, $k_{\text{hyd}}^{\text{CI}}=0$, panel C, ordered phosphorylation persists, showing that hydrolysis in the CI domain is not essential for generating a cycle at the level of the individual hexamers. If we only remove hydrolysis in CII and not in CI, there is still a small but clear cycle in state space due to the orchestrated switching between the active and inactive state caused by the phosphorylation state, and its effect on the affinity of the CII domain for KaiA (See S1 Fig in the supporting information). Clearly, ATP hydrolysis in CI or CII is necessary to generate a cycle. Yet, it is not sufficient: If we remove differential affinity of KaiA binding to the CII domain but keep ATP hydrolysis in CI and CII, $\delta g_{\text{bind}}^{\text{CII}\cdot \text{KaiA}}\left(X_{i}\right)=0$ and $\Delta G_{A,I}^{\text{CII}\cdot \text{KaiA}}=0$, all fluxes disappear in phosphorylation state space, as shown in Fig 8D. Hence, both differential affinity and ATP hydrolysis, most notably in the CII domain, are necessary to generate a phosphorylation cycle at the level of the individual hexamers.

### Results on KaiC ATPase and cycle dynamics

To test our model of the CI domain and to find the correct parameter values, shown in Table 3, we compare with the experiments on the evolution of the ATP fraction in the binding pockets of KaiC and the ATPase rate of KaiC [25, 26, 28, 39]. As is done in these experiments, we will study the behavior of different combinations of the main actors: KaiA, KaiB and KaiC and the ATP fraction *α*_ATP_. First we will study a system containing only KaiC, which allows us to constrain the parameters setting the rate of the hydrolysis cycle in the CI domain. Next, we look at the effect of dephosphorylation on the transient ATP fractions in the CI and CII domains, which provides an informative and testable prediction for how the CII domain regulates the ATP fraction in CI, which is indeed one of the key characteristics of our model. Then we study the effect of KaiA on the steady state ATPase rate and the dynamics of the ATP fraction in phosphorylating KaiC. For the full oscillating system, we will study the phase difference between the phosphorylation level and ATPase rate. Then we study whether the oscillations are robust to changes in the ATP fraction in solution, and whether our model can reproduce the experimental observation that the period of the oscillation is insensitive to these changes. Again, all simulations were performed with 720 KaiC hexamers and 720 KaiA dimers corresponding to the experimental standard condition of a 0.6μM concentration in a volume of 2 cubic micron.

**Table 3:** Model parameters relating to the CI domain are introduced in the theory section on the power cycle and their values are motivated in the results section. Energies are given in units of kT, were k is Boltzmann’s constant and T the temperature. Note that the rates of binding and unbinding of KaiA are for the CI domain. For rates relating to the CII domain, see Table 2.

Parameters relating to the CI domain		
Parameter	Value	Explanation
**Nucleotide binding pocket**		
$k_{\text{hyd}}^{\text{CI}}$	1.00 h^−1^	ATP hydrolysis in CI domain
$k_{\text{off}}^{\text{CI}\cdot \text{ADP}}$	1.50 h^−1^	ADP off-rate in CI domain
$\delta g_{\text{act},A}^{\text{CI}\cdot \text{ADP}}\left(U\right)$	0.00 kT	Activation energy contributions from the respective monomers, in the active state.
$\delta g_{\text{act},A}^{\text{CI}\cdot \text{ADP}}\left(T\right)$	-0.80 kT	
$\delta g_{\text{act},A}^{\text{CI}\cdot \text{ADP}}\left(D\right)$	0.40 kT	
$\delta g_{\text{act},A}^{\text{CI}\cdot \text{ADP}}\left(S\right)$	0.80 kT	
$\delta g_{\text{act},I}^{\text{CI}\cdot \text{ADP}}\left(U\right)$	-0.20 kT	Activation energy contributions from the respective monomer, in the inactive state.
$\delta g_{\text{act},I}^{\text{CI}\cdot \text{ADP}}\left(T\right)$	-0.80 kT	
$\delta g_{\text{act},I}^{\text{CI}\cdot \text{ADP}}\left(D\right)$	0.40 kT	
$\delta g_{\text{act},I}^{\text{CI}\cdot \text{ADP}}\left(S\right)$	0.80 kT	
**KaiA and KaiB sequestration dynamics**		
$k_{\text{on},A}^{\text{CI}\cdot \text{KaiA}}$	1.00 ⋅10^6^ μMh^−1^	KaiA on-rate, active
$k_{\text{off},A}^{\text{CI}\cdot \text{KaiA}}$	1.00 ⋅10^1^ h^−1^	KaiA off-rate, active
$k_{\text{on},I}^{\text{CI}\cdot \text{KaiA}}$	1.00 ⋅10^6^ μMh^−1^	KaiA on-rate, inactive
$k_{\text{off},I}^{\text{CI}\cdot \text{KaiA}}$	1.00 ⋅10^−1^ h^−1^	KaiA off-rate, inactive
$k_{\text{on},A}^{\text{CI}\cdot \text{KaiB}}$	1.00 ⋅10^−1^ h^−1^	KaiB on-rate, active
$k_{\text{off},A}^{\text{CI}\cdot \text{KaiB}}$	1.00 ⋅10^1^ h^−1^	KaiB off-rate, active
$k_{\text{on},I}^{\text{CI}\cdot \text{KaiB}}$	2.00 ⋅10^0^ h^−1^	KaiB on-rate, inactive
$k_{\text{off},I}^{\text{CI}\cdot \text{KaiB}}$	1.00 ⋅10^−2^ h^−1^	KaiB off-rate, inactive
$n_{\text{max}}^{\text{CI}\cdot \text{KaiA}}$	6	#KaiA sequestered/hexamer
$n_{\text{max}}^{\text{CI}\cdot \text{KaiB}}$	6	#KaiB sequestered/hexamer
**Conformational state**		
$k_{0}^{\text{conf}}$	10 h^−1^	prefactor conformational switch Offset energy in conformation A,I ADP dependent energy
$n_{0}^{\text{CI}\cdot \text{ADP}}$	5	
$\delta g_{A,I}^{\text{ATP},\text{ADP}}$	19 kT	

#### Ensemble of dephosphorylating KaiC shows a transient decrease in ATPase rate

To constrain the hydrolysis rate constant and the ADP dissociation rate in the CI domain of unphosphorylated KaiC, we used the experimental observation that in a system with only KaiC that has reached steady state, the ATP fraction in the binding pockets is around 30% [39] and the ATPase rate is 0.6 ATP per KaiC monomer per hour [25]. As we argued in the theory section on KaiA acting as a nucleotide exchange factor, without KaiA, the CII binding pockets are predominantly occupied by ADP, the monomers are in the U state, and the ATPase activity comes mainly from hydrolysis in the CI domain. Now, since the total fraction of ATP in the binding pockets is given by $0.5(\beta_{\text{ATP}}^{\text{CI}}$+$\beta_{\text{ATP}}^{\text{CII}})=0.3$, where $\beta_{\text{ATP}}^{\text{CI}}$ and $\beta_{\text{ATP}}^{\text{CII}}$ are the ATP fractions in the CI and CII domain, respectively, we estimate that the fraction of ATP in the CI domain, $\beta_{\text{ATP}}^{\text{CI}}=0.6$, because $\beta_{\text{ATP}}^{\text{CII}}\approx 0$. Assuming the measured ATPase rate equals the hydrolysis rate constant times the fraction of ATP in the CI binding pocket, $k_{\text{hyd}}^{\text{CI}}$ $\beta_{\text{ATP}}^{\text{CI}}$, we estimate that $k_{\text{hyd}}^{\text{CI}}=1.0{\text{ h}}^{-1}$. Since $\beta_{\text{ATP}}^{\text{CI}}=k_{\text{off}}^{\text{CI}\cdot \text{ADP}}/\left(k_{\text{off}}^{\text{CI}\cdot \text{ADP}}+k_{\text{hyd}}^{\text{CI}}\right)$, we deduce that $k_{\text{off}}^{\text{CI}\cdot \text{ADP}}=1.5{\text{ h}}^{-1}$.

**Fig 9:**
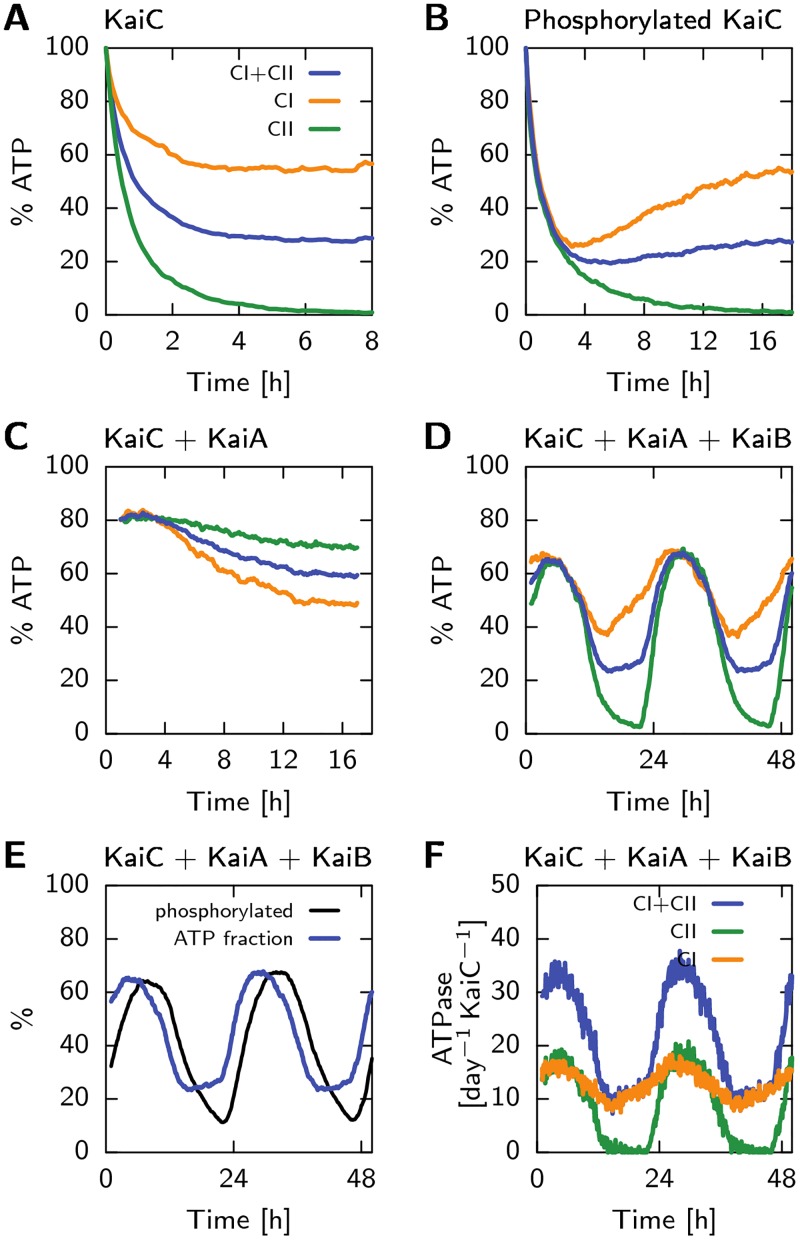
Model correctly predicts the ATP fractions in KaiC nucleotide binding pockets and the phase difference between this fraction and the phosphorylation level. Figures A-D show the fraction ATP/(ATP+ADP) in the nucleotide binding pockets of the CI domain (orange), the CII domain (green) and their sum (blue), in a 100% ATP solution, for different scenarios: KaiC initially unphosphorylated, no KaiA and KaiB present (A), KaiC initially phosphorylated, no KaiA and KaiB (B), initially unphosphorylated KaiC + KaiA (C) and KaiC + KaiA + KaiB (D). (A) The ATP levels drop monotonically due to the slow hydrolysis in both the CI and CII domain. (B) ATP fractions in dephosphorylating KaiC shows a clear trough in the ATP fraction of the CI domain due to the peak in the number of monomers in the S state (Fig 6A), which temporarily decreases the ADP-off-rate in the CI domain. (C) For a system with KaiC and KaiA, the ATP fraction is higher in the CII domain and lower in the CI domain. KaiA increases the nucleotide exchange rate in the CII domain, and the resultant high phosphorylation level decreases the ADP dissociation rate in CI, which decreases the ATP fraction in this domain. (D) Full system with KaiA, KaiB and KaiC shows oscillations in the ATP fractions of both the CI and CII domains. (E) The phase difference and amplitude of the ATP fraction (% ATP, blue line) and the phosphorylation level (% phosphorylated monomers, black line) agree well with experimental results in [39]. (F) ATPase levels of the KaiC domains show oscillations proportional to the ATP fractions in the respective domains.

To find out if an ensemble of only KaiC hexamers has the observed dynamics of the ATP fraction, we first consider a system in which KaiC is unphosphorylated and the ATP fraction in the binding pockets is 100%. We then study its relaxation to steady state. Fig 9A shows an exponential decay of the ATP fraction in both domains, on a similar timescale and steady state value as was found in [28]. The mean ATP fraction and ATPase rate, given in Table 4, are in quantitative agreement with experimental data presented above.

**Table 4:** Measured ATPase activity in ADP molecules produced per KaiC monomer per day (24 hours), under different conditions. First rows show results for parameters given in Tables 2 and 3. The last four rows show results for alternative models. When $k_{\text{hyd}}^{\text{CII}\cdot \text{KaiA}}=0$, the ATP hydrolysis in the CII domain is blocked when KaiA is bound to CII. When, $K_{\text{ATP}/\text{ADP}}^{\text{CII}}=10$, the CII domain has a higher relative affinity for ADP when the monomer is in the D state. The experimental values for the combined ATPase activity of the CI and CII domain, given in the last column, are taken from [25], and are shown for comparison.

Mixture			〈ATPase〉 [#ADP/KaiC/day]			
KaiA	KaiB	Condition	CI	CII	CI+CII	From [25]
-	-		14.4	0.0	14.4	14.5
+	-		11.5	17.0	28.5	18.1
-	+		14.2	0.0	14.2	8.9
+	+		12.3	8.8	21.1	15.8
+	+	*α*_ATP_ = 50%	13.0	8.3	21.3	-
+	-	$k_{\text{hyd}}^{\text{CII}\cdot \text{KaiA}}=0$	10.8	4.2	15.0	-
+	+	$k_{\text{hyd}}^{\text{CII}\cdot \text{KaiA}}=0$	13.9	3.0	16.9	-
+	-	$K_{\text{ATP}/\text{ADP}}^{\text{CII}}(\text{D})=10$	11.2	11.4	22.6	-
+	+	$K_{\text{ATP}/\text{ADP}}^{\text{CII}}(\text{D})=10$	12.8	7.4	20.2	-

Next we want to find out how the ATP fraction evolves in a system in which KaiC is initially highly phosphorylated, with the concentrations of monomers in the U,T,D and S state evolving as shown in Fig 6A. Please note that in our model, the T state will lower the activation energy for ADP dissociation set by $\delta g_{\text{barrier},A/I}^{\text{CI}\cdot \text{ADP}}\left(X_{i}\right)$, while the D and S state will increase the activation energy.

The parameter values for the contributions to the activation energy, $\delta g_{\text{barrier},A/I}^{\text{CI}\cdot \text{ADP}}\left(X_{i}\right)$, given in Table 3, were chosen to give good agreement of the oscillation dynamics presented below. We predict that the peak in the number of monomers in the S state (Fig 6A), which will decrease the ADP dissociation rate, causes a transient lowering of the overall bound ATP fraction, particularly in the CI domain. Indeed, Fig 9B shows a clear trough in the fraction of ATP in the binding pockets, which is most pronounced for CI. An experiment tracking the ATP fraction in the binding pockets, as performed in [39], but now starting with phosphorylated monomers, would be able to verify this prediction. Lastly, in our model the ATPase rate is proportional to the fraction of ATP in the binding pockets. Our model thus predicts a transient dip in the ATPase rate for dephosphorylating KaiC hexamers.

#### KaiA has opposite effects on the ATP fraction in the CI and CII domains

Adding KaiA to an ensemble of KaiC will immediately increase the ATP fraction in the CII binding pockets, causing phosphorylation of KaiC as shown in Fig 6B. Due to the phosphorylation of KaiC, the ATP fraction in the CI domain will decrease, because the ADP release in CI depends on the phosphorylation state of CII. These results are illustrated in Fig 9C, which also shows that the total ATP fraction in CI and CII stabilizes around 60%, in good agreement with [39]. However, the steady state ATPase rate of the ensemble is around 29 ADP molecules produced per monomer per day (ADP/KaiC/day), Table 4, higher than the observed rate of 18 ADP/KaiC/day [25]. This descrepancy could be due to our assumption that the ATP hydrolysis rate is constant, independent of both the phosphorylation state and whether or not KaiA is bound, and/or the assumption that the nucleotide affinities in the CII domain are independent of the phosphorylation state. It is conceivable that KaiA, when bound to CII, decreases the ATP hydrolysis rate in the CII domain. When we set the hydrolysis rate to zero when KaiA is bound, $k_{\text{hyd}}^{\text{CII}\cdot \text{KaiA}}=0$, and adjust the ADP dissociation rate to keep the phosphorylation dynamics unchanged, $k_{\text{off},\text{KaiA}}^{\text{CII}\cdot \text{ADP}}=0.2$, we find that the steady state ATPase rate in the system with only KaiA and KaiC drops to 15 ADP/KaiC/day (see Table 4), in good agreement with experiment. Another possibility is indeed that the affinity of doubly phosphorylated KaiC for ATP is lower than assumed in our model. In our current model, doubly phosphorylated KaiC has the same high affinity for ATP as KaiC in the other phosphorylation states (see Fig 3). When only KaiA is present and KaiC is often in the doubly phosphorylated D state, this sets up a futile cycle, in which KaiC continually binds ATP and then hydrolyzes it. Decreasing the relative affinity of D for ATP versus ADP, such that $K_{\text{ATP}/\text{ADP}}^{\text{CII}}=10$, lowers the steady state ATPase rate to about 22 ADP/KaiC/day. By making minor adjustments to parameters of the model, our model can thus reproduce the ATPase rate of KaiC in the presence of KaiA.

#### Delayed KaiA sequestration synchronizes the hexamers

Given our analysis in the results section on the phosphorylation dynamics, which showed that KaiC goes through a ordered phosphorylation cycle in solution with KaiA, we wanted to know if the sequestration of KaiA, after the slow binding of KaiB, will synchronize the KaiC hexamers. To this end we chose rates for KaiB binding and unbinding as presented in Table 3, which correspond to a very low affinity for KaiC in the active state, where KaiB is almost never bound, and a high affinity for the inactive state where KaiB binds and unbinds slowly. KaiA binds rapidly to the inactive CI domain when 6 KaiB monomers are bound to it, and dissociates from this complex very slowly. The last important quantity relating to the CI domain, which determines the stabilization of the inactive state of KaiC by ADP in the CI domain, $\delta g_{A,I}^{\text{ATP},\text{ADP}}$, is constrained from below by the relative affinities of KaiB and KaiA for the inactive state compared to the active state, as discussed in the theory section on the power cycle. Given the parameters for KaiA and KaiB binding to the CI domain in Table 3, we choose $\delta g_{A,I}^{\text{ATP},\text{ADP}}=19\text{ kT}$.

Fig 9D, 9E and 9F show clear oscillations in the ATP fraction in the CI and CII binding pockets, the phosphorylation fraction and the ATPase rates of the Kai oscillator, respectively. Panel E shows that the phase of the phosphorylation fraction is a few hours ahead of the ATP fraction in the binding pockets, which is in good agreement with experiments [39]. Also the amplitudes of both oscillations are in good agreement. The average ATPase rate of the oscillator is about 21 ADP/KaiC/day, which is slightly higher than the observed rate of 15 ADP/KaiC/day. We hypothesize that this high ATPase activity has to be attributed to the ATP hydrolysis in the CII domain. To test this, we set, as in the previous section, the hydrolysis rate constant to zero, $k_{\text{hyd}}^{\text{CII}\cdot \text{KaiA}}=0.0$, when KaiA is bound to the CII domain. In order for the phosphorylation dynamics to be comparable to our original model, we set $k_{\text{off},\text{KaiA}}^{\text{CII}\cdot \text{ADP}}=0.2$. In this model, the average ATPase activity has dropped to 16 ADP/KaiC/day, almost equal to the experimentally observed value (Table 4). Decreasing the affinity of doubly phosphorylated KaiC for ATP, which strongly reduced the ATPase rate of KaiC in the presence of KaiA only (see previous section), has a much smaller effect when both KaiA and KaiB are present, lowering the ATPase rate to 20 ADP/KaiC/day.

#### Clock period independent of bulk ATP fraction

**Fig 10:**
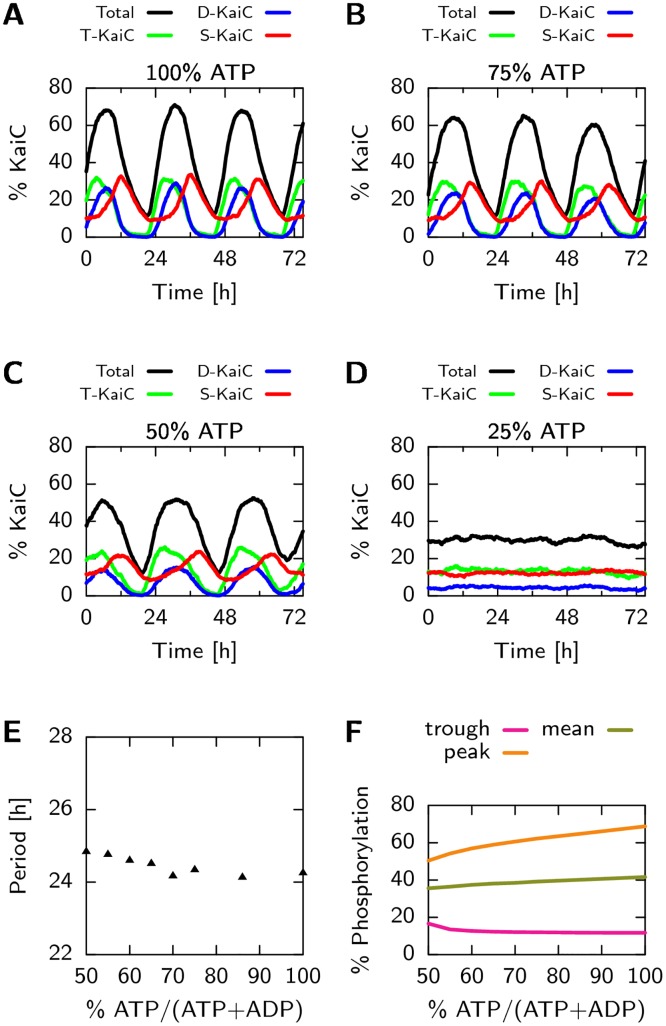
The Kai system oscillates over a wide range of ATP fractions, while the period remains unchanged. (A-D) Time traces of monomers in the T state (green), D state (blue), S state (red) and total phosphorylation level (black), for different ATP fractions in the bulk. Amplitudes and phases in good agreement with experiment (Compare with [27], S1 Fig), except that our system does not oscillate at 25% ATP fractions. (E) The average peak-to-peak time of the phosphorylation fraction for a 1000 hour time trace at different ATP fractions. The period is remarkably unaffected by the ATP fraction, even though it has a big influence on the phosphorylation speed, as we showed in Fig 6D. (F) Mean peak and trough phosphorylation levels at different ATP fractions. The increase in peak-hight with *α*_ATP_ is in in good agreement with experiment. However, in our simulations, the level of the trough is almost independent of *α*_ATP_, while in experiments it increases with *α*_ATP_ (Compare with Fig. S6 in [53] and Fig. 1 in [27]).

To find out how robust our model of the Kai oscillator is against changes in the steady-state ATP level, we simulate the oscillator at different ATP fractions, *α*_ATP_, as was done experimentally in [27]. Fig 10A, 10B and 10C show that the time traces of concentrations of monomers in the T,D and S state with *α*_ATP_ = 1.0,0.75 and 0.50, respectively, are in good quantitative agreement with experiments. Both the amplitude of the concentrations and their relative phases agree. However, contrary to experiments, at *α*_ATP_ = 0.25 the ensemble does not oscillate anymore. The reason is that not enough hexamers bind 6 KaiB monomers to sequester all the KaiA, as discussed in more detail in the Discussion. Since the absence of KaiA normally synchronizes the phosphorylation state of all hexamers, now the ensemble quickly becomes unsynchronized, and the oscillations disappear. Interestingly, in the range of ATP fractions where the Kai system does oscillate, the period of the oscillations is independent of *α*_ATP_, as is shown in panel E, and experimentally observed in [53]. This is remarkable, because we did not design the system to have a period independent of *α*_ATP_. In panel F we plot the peak, trough and mean phosphorylation level of the oscillations at different values of *α*_ATP_. The increase in peak height with *α*_ATP_ is in good agreement with experiments in [27]. However, while in our simulations the level of the troughs only marginally rises as *α*_ATP_ decreases, experiments show a considerable increase. Because in our simulations the appearance of free KaiA at the end of the cycle always occurs at a fixed phosphorylation ratio, it is hard to explain this discrepancy with experiments.

#### What is the principal driver of the oscillations: Hydrolysis in the CI or the CII domain?

In our model, there are two reactions that break detailed balance and allow the system to oscillate: Hydrolysis of ATP in the CI and in the CII domain. In the results section on phosphorylation dynamics we showed that hydrolysis of ATP in CII, in combination with differential affinity, is sufficient to generate cycles of phosphorylation at the level of the individual hexamers. However, without hydrolysis of ATP in the CI domain, the macroscopic oscillations inevitably come to a halt, because not enough KaiC can reach the inactive conformational state to allow for the necessary level of periodic KaiA sequestration. Clearly, while ATP hydrolysis in CI is not essential for generating cycles at the level of individual hexamers, it is necessary for generating macroscopic oscillations. The question that remains is whether hydrolysis of ATP in CII is likewise necessary for creating coherent, macroscopic oscillations. In the model presented so far, hydrolysis of ATP in CII is needed to allow for dephosphorylation: During the dephosphorylation phase, when a KaiC protein has made the transition from the D to the S state, it has ATP in the binding pocket, which needs to be hydrolyzed to generate ADP, thereby enabling the transition from S to U. However, this ATP hydrolysis reaction does not seem of fundamental importance for breaking detailed balance and creating macroscopic oscillations. Could this system generate oscillations with only turnover of ATP in the CI domain? To address this question, we here investigate a slightly modified version of our original model that has no ATP hydrolysis in the CII domain. This modified version does not represent the real Kai system, but rather serves as a thought experiment to clarify the different thermodynamic roles of ATP hydrolysis in the two domains.

We change our existing model so that KaiC can dephosphorylate without hydrolysis in the CII domain while still having KaiA stimulated phosphorylation in order to synchronize the hexamers. To this end, we set the ATPase rate in the CII domain to zero, while still allowing phosphates to be transferred in both directions between ATP (or ADP) and the serine and threonine residues. For dephosphorylation to occur, there must then be some mechanism other than ATP hydrolysis to introduce ADP’s into the CII binding pocket to receive the phosphates. We thus make the nucleotide exchange rate high in the inactive state. In the active state, however, the nucleotide exchange rate should still be low unless KaiA is bound to CII, to preserve the mechanism of KaiA stimulated phosphorylation. To maintain the correct relative stability of the two KaiC conformations, ADP in the CII domain now also stabilizes the inactive state, such that ADP has a high affinity for the CII domain when the hexamer is in the inactive state, ${\tilde{K}}_{\text{ATP}/\text{ADP}}^{\text{CII}}=10.0$, and the original low relative affinity for ADP when in the active state $K_{\text{ATP}/\text{ADP}}^{\text{CII}}=0.10$. Finally, because ADP from the bulk is required for dephosphorylation, we set *α*_ATP_ = 0.5. Changed parameters are listed in Table 5 below.

**Table 5:** Parameters used in the alternative model without hydrolysis in the CII domain that are different from the values in our original model in Tables 2 and 3. Parameters values are listed for both the active and inactive conformations if they have been changed for either conformation.

Parameter	Active	Inactive
$\begin{array}{lll} k_{\text{hyd}}^{\text{CII}} & & {(\text{h}}^{-\text{1}}\text{)} \end{array}$	0	0
$K_{\text{ATP}/\text{ADP}}^{\text{CII}}$	0.1	10
$\begin{array}{lll} k_{\text{off},0}^{\text{CII}\cdot \text{ATP}} & & \left({\text{h}}^{-1}\right) \end{array}$	0.6	6.0
$\begin{array}{lll} k_{\text{off},\text{KaiA}}^{\text{CII}\cdot \text{ATP}} & & \left({\text{h}}^{-1}\right) \end{array}$	6.0	6.0
$\begin{array}{lll} g_{\text{A},\text{I}}^{\text{ATP}/\text{ADP}} & & \left(\text{kT}\right) \end{array}$	30	30

**Fig 11:**
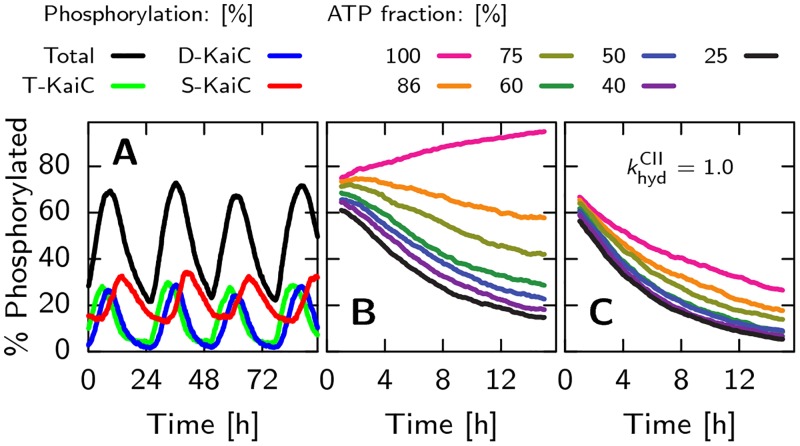
An alternative model, without hydrolysis in the CII domain, can generate robust macroscopic oscillations. (A) Time traces of monomers in the T state (green), D state (blue), S state (red) and total phosphorylation level (black), at 50% ATP level in the bulk, for the alternative model with hydrolysis only in the CI domain. (B) Dephosphorylation of KaiC for different bulk fractions of ATP, *α*_ATP_. Due to the high nucleotide exchange rate of the CII domain in the inactive conformation, the dephosphorylation speed becomes sensitive to *α*_ATP_, contrary to what is observed experimentally. (C) Even when we add the hydrolysis of ATP in the CII domain, $k_{\text{hyd}}^{\text{CII}}=1$, the speed still depends on *α*_ATP_.

Our modified model shows robust macroscopic oscillations, as shown in Fig 11A. The total ATP consumption has dropped to 11.6 ATP/KaiC/day, all due to the CI domain’s ATPase activity. This shows that the Kai oscillator can in principal generate macroscopic oscillations with only hydrolysis in the CI domain, and that hydrolysis in CII is not essential. However, as shown in Fig 11B, the rate of dephosphorylation now strongly depends on the ATP fraction in the bulk, because *α*_ATP_ affects the probability that, upon the D→S transition and subsequent ATP release, CII will bind ADP instead of ATP, which is necessary for the next S→U transition. This dependence of the dephosphorylation rate on the bulk ATP fraction in this modified model is contrary to what is observed in experiments. Even when we allow for hydrolysis in the CII domain in addition to nucleotide exchange in the inactive state, Fig 11C, the dephosphorylation speed still strongly depends on *α*_ATP_. As argued in [27], such a dependence of the dephosphorylation rate on *α*_ATP_ would hamper input compensation, and the period of the oscillations would not be constant any more at different ATP fractions. A low nucleotide exchange rate in the inactive state (as included in our main model described in the preceding sections) seems therefore critically important for the real Kai oscillator.

## Discussion

We set out to develop a thermodynamically correct model of the post-translational Kai oscillator that is consistent with the large body of quantitative experimental data available. In particular, the recent experimental observation that KaiC regenerates ATP during dephosphorylation [28, 29] made us rethink the thermodynamics behind the phosphorylation cycle: If phosphorylation and dephosphorylation require no net turnover of ATP, what drives the thermodynamic cycle of the oscillator? We built our new model on our earlier model of the Kai system [9, 16], where each individual KaiC hexamer goes through a cycle of phosphorylation and dephosphorylation. The hexamers are synchronized through the mechanism of differential affinity, in which the affinity of KaiA for KaiC changes with the phosphorylation state and the complex of inactive KaiC with KaiB has the strongest affinity for KaiA; sequestration of KaiA by this complex allows the hexamers lagging behind and which are still in the dephosphorylation phase of the cycle to remove KaiA from the front runners, which have already completed their cycle yet need KaiA to be phosphorylated again. Here, we have extended these ideas with a more detailed monomer model, taking into account the two phosphorylation sites per monomer and the nucleotide binding pockets in the CI and CII domain. This allowed us to include the effects of the ordered phosphorylation cycle on the monomer level, found in [8, 27].

### Summary of the key results of the model

Here we give an overview of the most important conclusions we can draw from our model.

#### By enhancing nucleotide exchange, KaiA both stimulates phosphorylation and blocks dephosphorylation, preventing futile cycles

In our new model, next to the threonine and serine phosphorylation sites, we explicitly track whether there is ATP or ADP present in the nucleotide binding pockets of the CI and CII domains of KaiC. Dephosphorylation proceeds exclusively by phosphotransfer between the phosphorylation site and ADP in the CII domain; direct release of inorganic phosphate from serine or threonine residues to the bulk can not occur. KaiA acts as a nucleotide exchange factor [39], enhancing the release rates of nucleotides from the CII domain. In combination with the hydrolysis of ATP in the CII domain, KaiA effectively increases the fraction of ATP in the binding pocket, thereby driving the hexamer towards the highly phosphorylated state. Because dephosphorylation with ATP in the binding pocket is impossible, KaiA, when bound, also effectively blocks dephosphorylation. In this way, the serine and threonine sites can be phosphorylated with a rate similar to the rate of dephosphorylation, because the phosphorylation rate does not need to compensate for the spontaneous release of phosphate groups. The capacity of KaiA to block dephosphorylation has two important consequences. First, if KaiA were not to block dephosphorylation, then the latter would inevitably occur, which must then be followed by another round of phosphorylation. Such futile cycles would make the oscillator less efficient. Furthermore, if KaiA were not to impede dephosphorylation, then *net* phosphorylation could only occur if the phosphorylation rate is larger than the dephosphorylation rate. Simulations reveal, however, that in such a model the concentration of threonine phosphorylated KaiC would rise too fast compared to the phosphorylation assay shown in Fig 6B. The timing of the peak of T state monomers would then be wrong.

#### Differential affinity of the CII domain for KaiA stimulates the ordered phosphorylation of the S and T sites

Next to regulating the ATP level in CII binding pockets, KaiA also steers the order of phosphorylation of the threonine and serine sites, which arises in our model as a logical consequence of differential affinity. In our model, KaiA has a higher affinity for KaiC that is in the T state, with threonine phosphorylated, than in the S state, with serine phosphorylated. Detailed balance then implies that the binding of KaiA raises the energy level of the S state compared to that of the T state (see the energy levels of Fig 3). In this way, the binding of KaiA to CII drives the ordered phosphorylation cycle, where first the threonine site is phosphorylated and then the serine site. We tested this with simulations, and found that KaiC hexamers in a solution with KaiA and no KaiB, go through the ordered phosphorylation cycle of the T and S sites. Hydrolysis of ATP bound to the CII domain is sufficient to drive this cycle. This prediction could conceivably be tested by performing mass-spectrometry experiments [8, 54], tracking the phosphorylation states starting from different initial conditions with synchronized hexamers, and testing whether the measured rates of phosphorylation and dephosphorylation obey detailed balance.

#### Binding multiple KaiA dimers to CII does not necessarily alter results

In our model, only a single KaiA dimer can bind to the CII domain to stimulate KaiC phosphorylation, while some experiments indicate that multiple KaiA dimers may bind to CII [48, 49]. Since the binding of multiple KaiA dimers to CII reduces the concentration of free KaiA, which could affect the mechanism of differential affinity, we checked whether such multiple binding significantly changes our results. To this end, we allowed two KaiA dimers to bind CII in a non-cooperative manner, each with the same affinity. We found that, when other parameters are adjusted appropriately to compensate, the timetraces of the phosphorylation level and the fraction of CII domains bound to KaiA are similar to those of the original model where only one KaiA can bind to CII (See S2 Fig in the supporting information). Because in our model KaiA rapidly binds and unbinds the CII domain, the net nucleotide exchange rate is proportional to the probability that KaiA is bound to KaiA, multiplied by the nucleotide-exchange rate given that KaiA is bound. As a result, the lower binding probability due to the lower free KaiA concentration resulting from the larger number of KaiA binding sites on CII can be compensated for by a higher exchange rate when KaiA is bound. Current experiments are not yet precise enough to make clear whether binding of multiple KaiA dimers to the CII domain, should it occur, is cooperative, so we do not consider that possibility here. As the experimental picture is refined, however, it will be interesting to explore whether cooperative binding can introduce additional effects, for example perhaps enhancing the oscillator’s robustness by allowing for sharper switching between the phosphorylation and dephosphorylation phases of the cycle.

#### ATP hydrolysis in CI drives conformational switching and macroscopic oscillations

Even though hydrolysis in the CII domain is sufficient to give rise to the phosphorylation cycle of the *individual* hexamers, we conclude that this is not the principal driver of the *macroscopic* oscillations, and in particular of the periodic sequestration of KaiA. If the phosphorylation of KaiC were to directly stabilize the inactive state and consequently the binding of KaiB, then detailed balance would dictate that, conversely, the latter also stabilize the phosphorylated state. Adding KaiB to a dephosphorylation assay would then decrease the dephosphorylation speed, contrary to what is found in experiments [8], which show no change in dephosphorylation dynamics. In our model, the phosphorylation cycle only sets the timing of the conformational switch by regulating the activation energy for ADP dissociation in the CI domain. Hydrolysis of ATP in the CI domain will continually generate ADP in the binding pocket of CI, but only when enough serine sites on CII have been phosphorylated, does the ADP release rate drop sufficiently so that the ADP level in CI will rise. It is this rise in ADP level that stabilizes the inactive state of KaiC. Hydrolysis in the CI domain thus drives the conformational switch and provides the large change in affinity between the active and inactive states, necessary for KaiB binding and KaiA sequestration. The latter, in turn, underlies the synchronization of the phosphorylation cycles of the individual hexamers, which is essential for generating the macroscopic oscillations in phosphorylation level.

#### Positive feedback is not essential; time delay and negative feedback are sufficient

Unlike in the model by Van Zon *et al*., here the phosphorylation dynamics of the hexamer is independent for each monomer, and a hexamer does not need to be fully phosphorylated before flipping to the inactive state. Phosphorylation of the threonine and serine sites in each KaiC monomer has antagonistic effects on the ADP dissociation rate from the CI domain, and consequently on the switch of the conformational state. Due to this antagonism, the conformational switch depends on the difference of T to S phosphorylated residues, and not on the absolute phosphorylation level. Therefore, in our model, a hexamer does not need to go through a full phosphorylation cycle each period, as was the case for the model by Van Zon and coworkers. Furthermore, there is no direct cooperativity between monomers; their states all add linearly to the activation energy for ADP dissociation in CI and the free energies of the conformational states. In particular, the D and S state have a similar effect on the ADP dissociation rate in CI, and hence on the conformation of the hexamer and on its ability to sequester KaiA. This means that the synchronization mechanism of the original monomer model by Rust et. al. [8] does not apply. In their model, KaiA prevents the occupation of the S state by enhancing the transition from the S to the D state, while only the S state sequesters KaiA. This mutual inhibition between KaiA and the S state creates a positive feedback loop for KaiA sequestration that is essential to the oscillations in that model. In contrast, in our model the D and S states both stimulate KaiA sequestration, so it does not exhibit this positive feedback mechanism. Because our model acts at the level of hexamers rather than monomers, it does not need the positive feedback: the delay between the conformational switch and the subsequent binding and sequestration of KaiA is sufficiently long that, together with the negative feedback of KaiA sequestration on phosphorylation, it can generate oscillations.

#### The model is robust to variations in ATP fraction

Our model correctly reproduces the phosphorylation and dephosphorylation time traces of the T,D and S state monomers. Furthermore, as in experiments [53], dephosphorylation is independent of the bulk ATP fraction, whereas phosphorylation does strongly depend on it. The idea that dephosphorylation is dominated by the phosphotransfer pathway [28, 29] is confirmed by good agreement with the experimentally observed ATP production in the CII domain and the delay in the appearance of inorganic phosphate in the bulk [28]. To compare the ATP consumption of KaiC in our model with experiments, we investigated the ATP fraction in the nucleotide binding pockets and the ATPase activity of KaiC, for different mixtures of Kai proteins. Both the transient dynamics of the ATP fraction in the binding pockets and the steady state ATPase rate in mixtures with KaiC only or KaiC and KaiA are in qualitative agreement with experiments. In a system with macroscopic oscillations, both the phase difference between the ATP fraction in the binding pockets and the phosphorylation fraction and the amplitude of the ATP fraction are in excellent agreement with experiments. To check the robustness of the oscillator against variations in the bulk ATP fraction, we checked whether oscillations persist at lower ATP fractions and if the phosphorylation period is independent of the fraction. We found that the clock period is indeed constant over a wide range of ATP fractions.

### Open questions

Although our model is able to reproduce most of the available experimental data, there are a few observations that it cannot replicate in its current form.

First, in our model, KaiB barely interacts with unphosphorylated KaiC, in contrast with the observation that KaiB lowers the ATPase activity of a solution with only KaiC [25], and that KaiB can bind unphosphorylated WT KaiC [56] at micromolar concentrations. In our model, it is essential that unphosphorylated KaiC is predominantly in the active conformation: Monomers in the U state increase the dissociation rate from ADP in the CI domain, thereby stabilizing the active state which leads to the dissociation of the sequestered KaiA and KaiB at the end of the cycle. The consequence is that unphosphorylated KaiC is predominantly in the active conformation, which has a very low affinity for KaiB. Making the inactive state of unphosphorylated KaiC more stable would remedy this shortcoming of the model, because in the inactive state KaiC can bind KaiB. Consistent with this idea, very recent experiments suggest that the inactive conformational state is indeed more stable: About half of the unphosphorylated KaiC hexamers in a system without KaiA or KaiB, are in the inactive conformational state [59]. However, increasing the affinity of KaiB for unphosphorylated KaiC also increases, in the current model, the capacity of unphosphorylated KaiC to sequester KaiA, which impedes the release of KaiA at the end of the cycle. For future research, it will be interesting to see whether by amending the model these experimental observations can be reproduced.

Secondly, the oscillations in our model come to a standstill when the bulk ATP fraction drops below 40%, while in experiments they continue to exist until the ATP fraction reaches 25% [27, 53]. There are two reasons why the oscillations come to a standstill when the ATP fraction is reduced sufficiently. The first is that a lower ATP fraction lowers the rate of phosphorylation, while the rate of dephosphorylation remains constant. This reduces the number of KaiC hexamers that make it to the top of the cycle during the delay between the moment KaiC reaches the top of the phosphorylation cycle (and no longer needs KaiA to progress along the cycle) and the moment KaiC sequesters KaiA (and the laggards that are still in the phosphorylation phase can no longer progress to the top). Because there are fewer hexamers that make it to the top of the cycle, fewer hexamers will later participate in sequestering KaiA, which shortens the time window in which KaiA is sequestered, weakening the synchronization mechanism. In addition, the interplay of a lower phosphorylation rate and a constant dephosphorylation rate causes the distribution of KaiC phosphoforms during the dephosphorylation phase to widen. This increases the likelihood that the front runners reach the bottom of the cycle before all KaiA is sequestered; the front runners will then be phosphorylated again, causing the synchronization mechanism to break down. These effects are exacerbated by the fact that at lower ATP fraction the individual hexamers traverse a smaller phosphorylation cycle, as discussed in a forthcoming publication [60]. While these are the principal mechanisms by which oscillations cease to exist at sufficiently low ATP concentrations, a question is whether there are mechanisms that can mitigate these effects. One possibility is to make the time window of sequestration more deterministic. In the model presented here the binding of KaiB and the subsequent sequestration of KaiA is not directly coupled to the phosphorylation state of KaiC, in contrast to the dynamics in the Van Zon model [9]. As a result, both the distribution of the delay till, and the window of, KaiA sequestration is rather broad. This decreases the likelihood that during dephosphorylation there will be a time window in which there are enough hexamers to sequester KaiA. It thus conceivable that making the window of KaiA sequestration more deterministic, e.g. by making dephosphorylation of the respective monomers within a hexamer more concerted or by more tightly coupling KaiB-KaiC binding to the KaiC phosphorylation state, extends the range of ATP concentration over which the model exhibits oscillations. We have not investigated the effects of the concerted phosphorylation of hexamers, because it deviates too much from our current model where monomers phosphorylate independently. Another way to enhance the sequestration capacity might be to increase the affinity of unphosphorylated KaiC for KaiB (and thus, indirectly, KaiA), but as mentioned above, this impairs the release of KaiA at the end of the cycle. Furthermore, it is conceivable that adding an explicit cooperativity term in Eq 10, describing the binding of ADP and KaiB to the CI domain, might make the oscillations more robust. Finally, it is also possible that including monomer exchange might improve synchronization of the KaiC hexamers and thus allow oscillations to persist to lower ATP fractions, but such an effect likewise cannot readily be included in the current model.

Lastly, the ATP consumption in our model is slightly higher than observed. We hypothesized that this can be attributed to the ATP hydrolysis in the CII domain. To provide support for this idea, we looked at an alternative model where the binding of KaiA suppresses ATP hydrolysis in CII. This model did show ATPase rates very similar to experimentally observed values.

### Predictions and experimental verification

Here we explore the possibilities for experimentally verifying the predictions from our model. Our model is based on two important ingredients: 1) The relative stability of the two conformations is determined by the ATP fraction in the binding pockets of the CI domain and 2) this fraction is set by the number of phosphorylated serine and threonine sites in the CII domain of the hexamer.

The dependence of the conformation on the nucleotide binding state of CI can be tested by measuring the ATP fraction in the binding pockets of KaiC [25, 39] while at the same time probing the conformational state as in the study of [59], where the authors track the fractions of active and inactive KaiC hexamers over time. Our analysis predicts a positive correlation between the fraction of ADP bound to CI and the fraction of inactive KaiC, including KaiC mutants with a different hydrolysis rate constant in CI [25] and in an assay where KaiC is in the presence of KaiA and KaiB, and oscillates over time.

The dependence of the ATP fraction in the binding pockets of the CI domain on the phosphorylation state of the CII domain can be tested by measuring the concentrations of monomers in the U,T,D and S phosphorylated state [8, 54] and again the ATP fraction in the nucleotide binding pockets [25, 39]. We predict that serine-phosphorylated KaiC slows down the dissociation of ADP from CI, decreasing its ATP fraction in the binding pocket, and threonine-phosphorylated KaiC should antagonize this effect. Starting with different ratios and levels of monomers phosphorylated at their threonine and serine sites, either using KaiC phosphomimics or aliquots from an oscillating system, the ADP fraction in the CI binding pockets should show a positive correlation with the *difference* between the number of phosphorylated serine sites and threonine sites. Adding KaiB should enhance the effect, because it cooperatively stabilizes the inactive state with ADP via the MWC mechanism. Our model can also explain the observation described in [61], where they found that adding ATP to unphosphorylated KaiC leads to a transient dip in the ATPase rate: The transient phosphorylation of KaiC temporarily lowers the CI-ADP dissociation rate. Clearly, it would be of interest to repeat these experiments starting with KaiC in different phosphorylation states, and in the presence and absence of KaiB. Our model predicts that, starting from fully phosphorylated KaiC, during dephosphorylation the ATP fraction in the binding pockets will exhibit a dip (see Fig 9).

Related to this, and more specifically, our model predicts that the ADP dissociation rate in the CI domain is set by the relative number of phosphorylated serine and threonine sites in the CII domain, and not by their absolute levels. This implies that not all the residues have to be phosphorylated before a hexamer can switch to the inactive conformation and complete its cycle. Indeed as we show in a forthcoming publication, at lower ATP fractions of the buffer, hexamers go through a smaller phosphorylation cycle. This could in principle be tested experimentally if it were possible to track individual hexamers as they go through their phosphorylation cycle.

While these experiments test our predictions on the connection between the CI and CII domain, our analysis also predicts an interesting consequence of the idea that phosphotransfer is the major pathway for dephosphorylation [28, 29]. This could be tested by revisiting the experiments on dephosphorylation of radioactively labeled KaiC [28], but now in solution with non-hydrolyzable ATP. Since the ATP can not be hydrolyzed, there will be no ADP in the CII binding pockets, and the phosphate groups on the S and T sites in KaiC can not be transferred to ADP in the CII domain. This should significantly slow down the dephosphorylation speed if indeed phosphotransfer is the dominant pathway.

**Fig 12:**
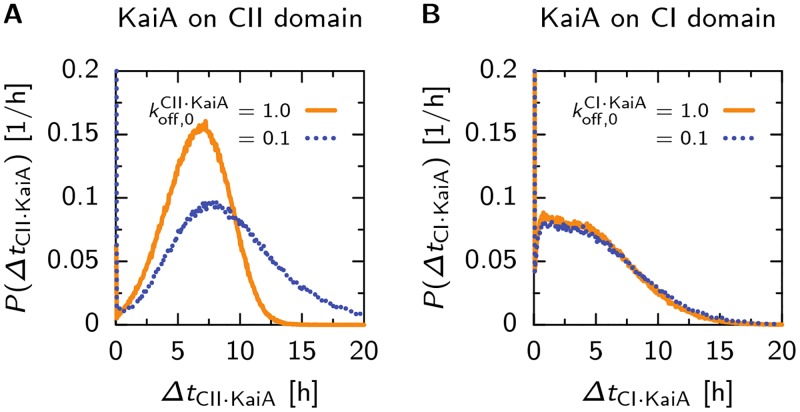
Probability density of the time, per period of the phosphorylation cycle, the CII domain of a KaiC hexamer is bound to KaiA, Δ*t*_CII·KaiA_. (A), or the CI domain has at least one KaiA dimer bound, Δ*t*_CI·KaiA_ (B). We compare situations with our standard value for the KaiA dissociation rate, $k_{\text{off},0}^{\text{CII}\cdot \text{KaiA}}=1.0$ (orange solid line) with a much lower value $k_{\text{off},0}^{\text{CII}\cdot \text{KaiA}}=0.1$ (blue dotted line). (A) For the CII domain, both values of the KaiA dissociation rate show unimodal distributions of the time KaiA is bound. This indicates that, during a period, KaiA binds to all hexamers in the ensemble equally likely. Of all hexamers, only 1% do not bind KaiA to CII at any time during the period. (B) For the CI domain, the time KaiA is sequestered by KaiC is roughly exponentially distributed, and is the same for both KaiA dissociation rates from the CII domain. Here, 30% of the hexamers do not sequester KaiA at all during a period, indicating many hexamers do not sequester KaiA during a cycle.

Our model of the interaction of KaiA with the CI and CII domains of KaiC also allows us to make predictions for how long KaiA is bound to one of the domains during an oscillation. In our model, the dissociation rate of KaiA from the CII domain is much higher than the frequency of the oscillation. This is essential for differential affinity, where KaiA continually binds different KaiC hexamers during the phosphorylation phase to promote the phosphorylation of hexamers that are lagging behind. Our model therefore predicts a sharp peak in the distribution of times for which the CII domain of a certain KaiC hexamer is bound to KaiA during an oscillation period, Δ*t*_CII·KaiA_. Indeed, Fig 12A shows a clear single peak in this distribution. When we lower the dissociation rate of KaiA from the CII domain, this distribution broadens, Fig 12B, indicating differential affinity is hampered. One might think that when the dissociation rate is decreased even further, the distribution in Δ*t*_CII·KaiA_ becomes bimodal, because once KaiA is bound to the CII domain of a certain hexamer, it will continue to stay bound to this hexamer during the whole phosphorylation phase of an oscillation cycle; because KaiA is limiting, this means that other KaiC hexamers will not, via their CII domain, bind KaiA during that cycle. In this case one fraction of hexamers does not or only very briefly binds KaiA via the CII domain while the other fraction is bound to KaiA for most of the time during the phosphorylation phase. However, in our model oscillations stop when we decrease the dissociation rate of KaiA to such a low level to allow for a bimodal distribution. Nevertheless, bi-modality could arise in experiments, which would indicate that the role of KaiA during the phosphorylation phase is very different from what we predict in our model. For KaiA bound to the CI domain, our simulations show exponentially distributed bound times, Fig 12B. This distribution is bimodal, as only 70% of the hexamers sequester KaiA, while the other 30% do not make it to the inactive state and bind six KaiB during an oscillation. Techniques to follow protein complex formation at the single molecule level have been developed [62], suggesting that future experiments might be able to reveal how long KaiA is bound to KaiC during an oscillation cycle.

## Methods

We model the post-translational Kai oscillator using kinetic Monte Carlo [63, 64]. All code, written in C++, is available at https://kmc-kaic.sourceforge.io. Each monomer has two nucleotide binding states for both the CI and CII domain and 4 phosphorylation states. Additionally, a hexamer can be in an active or inactive state. This results in (2 ⋅ 2 ⋅ 4)^6^ ⋅ 2 = 2^25^ different states a single hexamer can be in. Furthermore, there are the binding reactions with the KaiC hexamer: 2 for KaiA with the CII domain, 6 for KaiB monomers to the CI domain and another 6 reactions for KaiA binding to the CI domain of KaiC with 6 KaiB monomers. The total number of reactions therefore exceeds a billion. Clearly, the reaction combinatorics makes a straightforward ODE or Gillespie simulation unfeasible. Also the Quasi-Steady-State Approximation (QSSA) cannot be used, not only because of this combinatorial explosion of reactions, but also because most reactions do not exhibit a clear separation of timescales [65]. We thus set out to design a dedicated kinetic Monte Carlo algorithm to simulate the Kai system, where we do not have to write down all possible reactions explicitly.

To this end, we designed an algorithm that keeps track of *N*^hex^ KaiC hexamers, which in turn consist of six explicitly simulated monomers, and $N_{\text{tot}}^{\text{KaiA}}$ KaiA dimers. Since we have defined individual hexamers and monomers, we can calculate the propensity that a reaction occurs in a particular hexamer, and, in turn, the propensity that a reaction occurs in a monomer that is part of this hexamer. This allows us to create a layered version of the original algorithm by Gillespie [63], where we first determine the next reaction time and in which hexamer this reaction will take place. Then, in the next step, we determine which reaction or monomer of this hexamer fires. If a monomer fires, we choose which reaction of this monomer happens. This layered approach allows us to separate state changes in a hexamer that only modify the reaction propensities of that specific hexamer from state changes that influence the KaiA concentration in solution, which affects all hexamers. This greatly reduces the reaction combinatorics and the computational cost of the algorithm.

Specifically, the state of the whole system, s^tot^, consists of the state of the hexamers with index *h*, $s_{h}^{\text{hex}}$, and the number of KaiA dimers in solution, $n_{\text{sol}}^{\text{KaiA}}:s_{}^{\text{tot}}=\left\{{\left\{s_{h}^{\text{hex}}\right\}}_{h=1}^{N_{}^{\text{hex}}},n_{\text{sol}}^{\text{KaiA}}\right\}$. The hexamer state contains the state vectors of its six monomers with index *m*, $s_{h,m}^{\text{mon}}$, the conformational state, *C*, the number of KaiA bound to CI, *n*^CI·KaiA^, the number of KaiB bound to CI, *n*^CII·KaiA^, and the number of KaiA bound to the CII domain, *n*^CII·KaiA^: $s_{h}^{\text{hex}}={\left\{{\left\{s_{h,m}^{\text{mon}}\right\}}_{m=1}^{6},C,n_{}^{\text{CI}\cdot \text{KaiA}},n_{}^{\text{CI}\cdot \text{KaiB}},n_{}^{\text{CII}\cdot \text{KaiA}}\right\}}_{h}$. The state vector of a monomer consists of the threonine and serine phosphorylation site and the nucleotide binding pockets of the CI and CII domain: $s_{h,m}^{\text{mon}}={\left\{S,T,n_{\text{nucl}}^{\text{CI}},n_{\text{nucl}}^{\text{CII}}\right\}}_{h,m}$.

Given the system state, we can calculate the firing propensity of reaction *μ* that changes the state vector of monomer *m*, which is part of hexamer *h*, $q_{h,m}^{\mu }\left(s_{h}^{\text{hex}}\right)$. Note that this reaction propensity can depend on the state of the whole hexamer, and therefore is a function of the hexamer state vector, and not only of the state vector of monomer *m*. The propensity for firing a reaction with index *ν*, which changes the state variables of hexamer *h*, denoted $q_{h}^{\nu }\left(s_{h}^{\text{hex}},n_{\text{sol}}^{\text{KaiA}}\right)$, depends on the state vector of hexamer *h* only, and the number of KaiA in solution. Given these reaction propensities, we can calculate the accumulated propensities, denoted by $\tilde{q}$, of firing a single monomer *m* in hexamer *h*, a single hexamer *h* and the total propensity as

$$\begin{array}{ccc} {\tilde{q}}_{h,m}^{\text{mon}}\left(s_{h}^{\text{hex}}\right) & = & \sum_{\mu }q_{h,m}^{\mu }, \end{array}$$ (19)

$$\begin{array}{ccc} {\tilde{q}}_{h}^{\text{hex}}\left(s_{h}^{\text{hex}},n_{\text{sol}}^{\text{KaiA}}\right) & = & \sum_{m=1}^{6}{\tilde{q}}_{h,m}^{\text{mon}}+\sum_{\nu }q_{h}^{\nu }, \end{array}$$ (20)

$$\begin{array}{ccc} {\tilde{q}}_{}^{\text{tot}}\left({\left\{s_{}^{\text{hex}}\right\}}_{h=1}^{N_{}^{\text{hex}}},n_{\text{sol}}^{\text{KaiA}}\right) & = & \sum_{h=1}^{N_{}^{\text{hex}}}{\tilde{q}}_{h}^{\text{hex}}, \end{array}$$ (21)

respectively.

Since the firing of a reaction is a Markov process, the probability of hexamer *h* firing in the infinitesimal time interval [*t* + *τ*, *t* + *τ* + *dτ*] is

$$\begin{array}{c} P\left(\tau ,h|s_{}^{\text{tot}},t\right)d\tau ={\tilde{q}}_{h}^{\text{hex}}\;\text{exp}\left(-{\tilde{q}}_{}^{\text{tot}}\tau \right)d\tau . \end{array}$$ (22)

Now, given two random numbers, *ρ*_1_, *ρ*_2_, drawn from a uniform distribution with domain [0, 1), we calculate the next event time, *τ*, and the hexamer to fire, *h*, as

$$\begin{array}{ccc} \tau & = & \frac{1}{{\tilde{q}}_{}^{\text{tot}}}\text{ln}\left(\frac{1}{\rho_{1}}\right), \end{array}$$ (23)

$$\begin{array}{ccc} h & = & \text{the}\;\text{smallest}\;\text{integer}\;\text{satisfying}\;\sum_{h^{\prime }=1}^{h}{\tilde{q}}_{h}^{\text{hex}}>\rho_{2}{\tilde{q}}_{}^{\text{tot}}, \end{array}$$ (24)

respectively.

Having defined the important propensities, our dedicated kinetic Monte Carlo algorithm becomes

0 Initialize the time *t* = *t*_0_ and the system’s state $s_{}^{\text{tot}}=s_{0}^{\text{tot}}$. Calculate the propensities $q_{h,m}^{\mu }$, $q_{h}^{\nu }$, ${\tilde{q}}_{h,m}^{\text{mon}}$, ${\tilde{q}}_{h}^{\text{hex}}$ and ${\tilde{q}}_{}^{\text{tot}}$.
1 Calculate the time interval to the next reaction, *τ*, using Eq 23.
  - Choose which hexamer, *h*, to fire, with $P\left(h|\tau \right)={\tilde{q}}_{h}^{\text{hex}}/{\tilde{q}}_{}^{\text{tot}}$.
  - Choose which reaction, *ν*, with $P\left(\nu |\tau ,h\right)=q_{h}^{\nu }/{\tilde{q}}_{h}^{\text{hex}}$ or monomer, *m*, with $P\left(m|\tau ,h\right)=q_{h,m}^{\text{mon}}/{\tilde{q}}_{h}^{\text{hex}}$, to fire.
  - If a monomer was chosen, choose which reaction to fire with $P\left(\mu |\tau ,h,m\right)=q_{h,m}^{\mu }/{\tilde{q}}_{h,m}^{\text{mon}}$.
2 Fire reaction and update the state vector of hexamer *h*, and $n_{\text{sol}}^{\text{KaiA}}$ in case of a bimolecular reaction. Recalculate all reaction propensities $q_{h}^{\nu }$ and $q_{h,m}^{\mu }$ for each monomer *m*. In case a bimolecular reaction was fired, change $n_{\text{sol}}^{\text{KaiA}}$ accordingly, and update the bimolecular reaction propensities in all hexamers.
3 Recalculate ${\tilde{q}}_{h,m}^{\text{mon}}$ and ${\tilde{q}}_{h}^{\text{hex}}$ for the fired hexamer *h*. In case of a bimolecular reaction, also recalculate ${\tilde{q}}_{h^{\prime }}^{\text{hex}}$ for all hexamers *h*′. Update ${\tilde{q}}_{}^{\text{tot}}$.
4 Record ($t,s_{}^{\text{tot}}\left(t\right)$) as desired, return to step 1.

## Supporting information

S1 FigWithout hydrolysis in the CII domain, ordered phosphorylation remains.Hydrolysis in the CI domain, without KaiA sequestration by the CI domain and hydrolysis in the CII domain, is sufficient to generate the ordered phosphorylation of the T and S sites in a solution of KaiC and KaiA. Note that we lowered the bulk ATP fraction down to 25% to prevent the CII binding pockets from being occupied by ATP most of the time. Heatmaps of the probability, $P_{n_{T}^{},n_{S}^{}}$, for a single hexamer, of having *n*_T_ phosphorylated threonine sites and *n*_S_ phosphorylated serine sites. Arrows indicate the net flux through a state, where the length is proportional to the magnitude of the flux. Compared to Fig 8 of the main text, we amplified the arrows by a factor 5 to clearly show that a circular flux is present in the state space.(PDF)

S2 Fig Alternative model of two KaiA dimers binding to CII domain. Time traces of the phosphorylation level (black line) and the fraction of the CII domains of KaiC hexamers with at least one KaiA dimer bound (purple line), for a modified model where up to two KaiA dimers can bind to the CII domain of a hexamer (A) and for the original model where only one KaiA can bind to CII (B). In the modified model, the first and second dimer bind to the CII domain with the same affinity, such that there is no cooperative binding. Furthermore, the nucleotide exchange rate in the CII domain is already maximally enhanced when only one KaiA is bound. Clearly, the phosphorylation level time traces are qualitatively the same for both models. The fraction of hexamers having at least one KaiA bound on CII has a lower amplitude in the modified model, because some hexamers will have two KaiA dimers bound such that KaiA is less equally distributed as compared to the original model. To compensate for lower fraction of time KaiA is bound to the CII domain, we enhanced the nucleotide exchange rate in the CII domain when KaiA is bound by a factor of three.
(PDF)
