# Supplementary material for: A thermodynamically consistent model of the post-translational Kai circadian clock
Source: PLoS Comput Biol. 2017 Mar 15;13(3):e1005415. doi: 10.1371/journal.pcbi.1005415 (PMC5371392; doi:10.1371/journal.pcbi.1005415)
Supplement: S1 Fig — Hydrolysis in the CI domain, without KaiA sequestration by the CI domain and hydrolysis in the CII domain, is sufficient to generate the ordered phosphorylation of the T and S sites in a solution of KaiC and KaiA. Note that we lowered the bulk ATP fraction down to 25% to prevent the CII binding pockets from being occupied by ATP most of the time. Heatmaps of the probability, PnT,nS, for a single hexamer, of having nT phosphorylated threonine sites and nS phosphorylated serine sites. Arrows indicate the net flux through a state, where the length is proportional to the magnitude of the flux. Compared to Fig 8 of the main text, we amplified the arrows by a factor 5 to clearly show that a circular flux is present in the state space. (PDF) [file pcbi.1005415.s001.pdf]

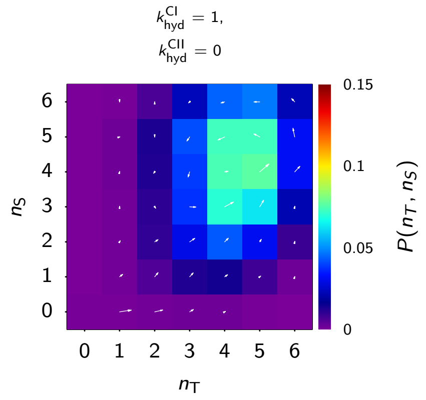

S1 Fig. Without hydrolysis in the CII domain, ordered phosphorylation remains. Hydrolysis in the CI domain, without KaiA sequestration by the CI domain and hydrolysis in the CII domain, is sufficient to generate the ordered phosphorylation of the T and S sites in a solution of KaiC and KaiA. Note that we lowered the bulk ATP fraction down to 25% to prevent the CII binding pockets from being occupied by ATP most of the time. Heatmaps of the probability, $P_{n_{\mathrm{T}},n_{\mathrm{S}}}$, for a single hexamer, of having n_{T} phosphorylated threonine sites and n_{S} phosphorylated serine sites. Arrows indicate the net flux through a state, where the length is proportional to the magnitude of the flux. Compared to Fig. 8 of the main text, we amplified the arrows by a factor 5 to clearly show that a circular flux is present in the state space.
