# Supplementary material for: A thermodynamically consistent model of the post-translational Kai circadian clock
Source: PLoS Comput Biol. 2017 Mar 15;13(3):e1005415. doi: 10.1371/journal.pcbi.1005415 (PMC5371392; doi:10.1371/journal.pcbi.1005415)
Supplement: S2 Fig — Time traces of the phosphorylation level (black line) and the fraction of the CII domains of KaiC hexamers with at least one KaiA dimer bound (purple line), for a modified model where up to two KaiA dimers can bind to the CII domain of a hexamer (A) and for the original model where only one KaiA can bind to CII (B). In the modified model, the first and second dimer bind to the CII domain with the same affinity, such that there is no cooperative binding. Furthermore, the nucleotide exchange rate in the CII domain is already maximally enhanced when only one KaiA is bound. Clearly, the phosphorylation level time traces are qualitatively the same for both models. The fraction of hexamers having at least one KaiA bound on CII has a lower amplitude in the modified model, because some hexamers will have two KaiA dimers bound such that KaiA is less equally distributed as compared to the original model. To compensate for lower fraction of time KaiA is bound to the CII domain, we enhanced the nucleotide exchange rate in the CII domain when KaiA is bound by a factor of three. (PDF) [file pcbi.1005415.s002.pdf]

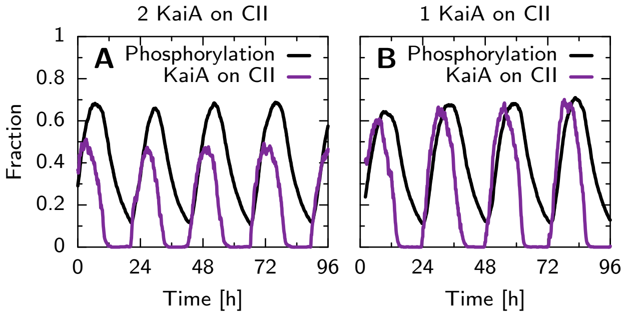

S2 Fig. Alternative model of two KaiA dimers binding to CII domain. Time traces of the phosphorylation level (black line) and the fraction of the CII domains of KaiC hexamers with at least one KaiA dimer bound (purple line), for a modified model where up to two KaiA dimers can bind to the CII domain of a hexamer (A) and for the original model where only one KaiA can bind to CII (B). In the modified model, the first and second dimer bind to the CII domain with the same affinity, such that there is no cooperative binding. Furthermore, the nucleotide exchange rate in the CII domain is already maximally enhanced when only one KaiA is bound. Clearly, the phosphorylation level time traces are qualitatively the same for both models. The fraction of hexamers having at least one KaiA bound on CII has a lower amplitude in the modified model, because some hexamers will have two KaiA dimers bound such that KaiA is less equally distributed as compared to the original model. To compensate for lower fraction of time KaiA is bound to the CII domain, we enhanced the nucleotide exchange rate in the CII domain when KaiA is bound by a factor of three.
